# Protein Palmitoylation in Bovine Ovarian Follicle

**DOI:** 10.3390/ijms222111757

**Published:** 2021-10-29

**Authors:** Svetlana Uzbekova, Ana-Paula Teixeira-Gomes, Aurélie Marestaing, Peggy Jarrier-Gaillard, Pascal Papillier, Ekaterina N. Shedova, Galina N. Singina, Rustem Uzbekov, Valerie Labas

**Affiliations:** 1 CNRS, IFCE, INRAE, Université de Tours, PRC, 37380 Nouzilly, France; marestaing.aurelie@hotmail.fr (A.M.); peggy.jarrier-gaillard@inrae.fr (P.J.-G.); pascal.papillier@inrae.fr (P.P.); valerie.labas@inrae.fr (V.L.); 2 INRAE, Université de Tours, ISP, 37380 Nouzilly, France; ana-paula.teixeira@inrae.fr; 3 L.K. Ernst Federal Research Center for Animal Husbandry, Dubrovitzy 60, 142132 Podolsk, Russia; shedvek@yandex.ru (E.N.S.); g_singina@mail.ru (G.N.S.); 4 Laboratoire Biologie Cellulaire et Microscopie Électronique, Faculté de Médecine, Université de Tours, 37032 Tours, France; rustem.uzbekov@univ-tours.fr

**Keywords:** protein palmitoylation, ZDHHC, granulosa, cumulus, oocyte, follicular fluid extracellular vesicles, bovine

## Abstract

Protein palmitoylation is a reversible post-translational modification by fatty acids (FA), mainly a palmitate (C16:0). Palmitoylation allows protein shuttling between the plasma membrane and cytosol to regulate protein stability, sorting and signaling activity and its deficiency leads to diseases. We aimed to characterize the palmitoyl-proteome of ovarian follicular cells and molecular machinery regulating protein palmitoylation within the follicle. For the first time, 84 palmitoylated proteins were identified from bovine granulosa cells (GC), cumulus cells (CC) and oocytes by acyl-biotin exchange proteomics. Of these, 32 were transmembrane proteins and 27 proteins were detected in bovine follicular fluid extracellular vesicles (ffEVs). Expression of palmitoylation and depalmitoylation enzymes as palmitoyltransferases (ZDHHCs), acylthioesterases (LYPLA1 and LYPLA2) and palmitoylthioesterases (PPT1 and PPT2) were analysed using transcriptome and proteome data in oocytes, CC and GC. By immunofluorescence, ZDHHC16, PPT1, PPT2 and LYPLA2 proteins were localized in GC, CC and oocyte. In oocyte and CC, abundance of palmitoylation-related enzymes significantly varied during oocyte maturation. These variations and the involvement of identified palmitoyl-proteins in oxidation-reduction processes, energy metabolism, protein localization, vesicle-mediated transport, response to stress, G-protein mediated and other signaling pathways suggests that protein palmitoylation may play important roles in oocyte maturation and ffEV-mediated communications within the follicle.

## 1. Introduction

Palmitic acid (C16:0) is the most abundant fatty acid (FA) produced in mammalian cells, which represents 32% of total FA content in bovine oocyte [1]. In human tissues, about 10–20% of proteins are modified by thioester linkage of deprotonated palmitate C16:0 to cysteine (Cys) in a process known as protein palmitoylation [2,3]. Protein palmitoylation is one of the major post-translational modifications (PTM) by lipids, which mediates the association of soluble proteins with membrane, their subcellular trafficking between membrane compartments and involved in protein-protein interactions, signaling and other intracellular processes determining protein localization, activity, and cell differentiation in different tissues [4,5,6]. Protein palmitoylation is an only reversible lipid modification, in contrast to other PTMs by lipids as N-terminal myristoylation and C-terminal isoprenylation, which also tether modified proteins to cytosolic membranes but remain constantly attached to the protein [4,7]. Palmitoylation regulates functional activity of many integral and peripheral membrane proteins involved in cell signaling, including G-proteins and their receptors (GPCRs) [8,9,10,11]. The role of protein palmitoylation in the cells has been extensively studied in cancer [12] and neuron diseases [13] because many of cancer-related proteins and neurotransmitter receptors are palmitoylated and deficiency in protein palmitoylation leads to diseases. In concordance, analyses of palmitoylomes in human diseases indicated a pivotal role of protein palmitoylation in nervous system disorders and cancers [14]. 

Development of methodological approaches of large-scale protein palmitoylation profiling in mammalian cells, which are based on acyl-biotin exchanges (ABE) method [15,16,17], allowed detection of palmitoylation of numerous soluble and membrane proteins in different tissues in human and other species, including bovine [5,10]. Several thousand proteins reported from palmitoyl-proteomes of different origin are referenced in the SwissPalm protein database (https://swisspalm.org/, accessed on 12 March 2021) [3]. In the list of palmitoyl-proteins, there are numerous G-proteins and GPCRs, secreted proteins, receptors of estrogen, growth factors, immune receptors and other proteins [9,18,19]. Many proteins, which were found palmitoylated in different tissues, are largely involved in female reproduction through their activity in the ovarian follicular cells and enclosed oocytes [20,21,22], although no data on palmitoylation of these proteins in the ovarian cells in mammals were reported yet. In Xenopus, palmitoylation of H-Ras protein and G-protein subunit alpha (GNAS) are involved in oocyte meiotic arrest [23,24]. In mice, point mutation of estrogen receptor alpha that precludes its palmitoylation and membrane trafficking leads to multiple abnormalities including infertility [25].

The mechanism of protein palmitoylation are extensively studied in human cells. Although some proteins are able to autopalmitoylate spontaneously [26], the majority of protein palmitoylation is catalyzed by membrane-associated palmitoyl-acyltransferases (PATs) [27]. Known PATs are zinc finger multipass transmembrane enzymes containing catalytic an Asp-His-His-Cys motif embedded within a cysteine-rich domain (ZDHHC), which catalyze acyl transfer from long chain fatty acyl-CoA to Cys residues [28]. ZDHHC PATs localize mainly to endoplasmic reticulum (ER), Golgi, and endosome membranes, but also to the plasma membranes [27,29]. In humans, 23 genes coding for ZDHHC proteins are known; and for several of them the PAT activity was experimentally confirmed with different substrate proteins in vivo and in vitro [30,31,32]. Expression of numerous *ZDHHC* genes is linked to cancer progression [12], and many proteins considered to be drivers of cancer are palmitoylated [33]. Mutations in *ZDHHC* genes are involved in pathogenesis of known neurological disorders as Huntington’s disease (*DHHC17*), schizophrenia (ZDHHC8) and X-linked mental retardation (*ZDHHC9* and *ZDHHC15*) [14,34]. 

Protein depalmitoylation is a part of palmitoylation dynamics and involves several enzymes, including two members of serine hydrolase family, acyl-protein thioesterases (APTs) LYPLA1 and LYPLA2 that cleaved palmitate from S-palmitoylated proteins [35]. In addition, palmitoyl-protein thioesterases PPT1 (encoded by *CLN1* gene) and its paralog PPT2 can remove thioester-linked fatty acyl groups from modified Cys residues in palmitoylated proteins or peptides during lysosomal degradation [35]. In addition, alpha-beta hydrolase-domain (ABHD) containing proteins as ABHD17 have emerged as novel potential depalmitoylating enzymes in mammals [27,35]. Repetition of palmitoylation/depalmitoylation cycles constitutes dynamic palmitoylation of cytosol and membrane proteins [10], and plays important roles in targeting proteins to membranes and interaction with lipid rafts, shuttling the proteins between cell compartments, thus regulating cell signaling. 

Reversible protein palmitoylation allows shuttling the proteins between cell compartments, including subcellular vesicle transport [36]. Significant enrichment of palmitoylated proteins was reported in the core complexes of caveolae and tetraspanin-enriched microdomains, and in lipid rafts [37], and in extracellular vesicles (EVs) [38]. In cancer cells, protein palmitoylation plays a role in formation of EVs in part by regulating proper localization and interaction of EV-specific tetraspanins [39], thus maintaining a proper membrane structure organization of exosome-like EVs [38]. Inhibition of protein palmitoylation affected number and size distribution of EVs released by muscle cells [38] and reduced localization of cancer-specific palmitoyl-proteins in EVs released from prostate cancer cells [39]. 

As any biological fluid, ovarian follicular fluid (FF) contains EVs of different types, which differ by their genesis, size and biological properties. Large/medium size EVs (defined as microvesicles, 100–1000 nm) formed by outward budding and fission of the plasma membrane. Small 30–150 nm EVs, also defined as exosomes, are formed within the endosomal network, and released from multivesicular bodies upon their fusion with the plasma membrane [40,41]. In bovine ovary, size and concentration of follicular fluid EVs (ffEVs) significantly varied between the follicles at different growth stages [42,43]. Small exosome-like ffEVs, which are released to follicular fluid by follicular cells and the oocyte, transport molecular cargo of RNAs, proteins and lipids [42,44,45], and are involved in molecular exchanges between an oocyte and surrounding cumulus cells (CC), and granulosa cells (GC) [46]. At the ovarian level, small ffEVs mediate molecular signaling in the follicle and affect oocyte competence to develop embryo [47]. Both vesicle-mediated and direct cell-to-cell molecular interactions between the oocyte and surrounding follicular cells are involved in the regulation of follicle growth [48], follicle dominance [49], and oocyte meiotic arrest [50]. Inhibition of ZDHHC3-directed palmitoylation in Xenopus egg resulted in a failure to maintain meiotic arrest [24]. In mammals, a role of protein palmitoylation in the ovarian follicular cells and oocyte remains to be elucidated. 

The objective of our study was to characterize, for the first time, protein palmitoylation machinery in bovine ovarian follicle. We have identified palmitoylated proteins from follicular GC and cumulus-oocyte complexes and looked for these proteins in ffEVs. Expression of specific ZDHHCs and depalmitoylation enzymes was analyzed in follicular GC, CC and oocytes, and then investigated in relation to oocyte maturation. 

## 2. Results

### 2.1. Isolation and Identification of Potentially Palmitoylated Proteins in Follicular Cells

**Figure A1:**
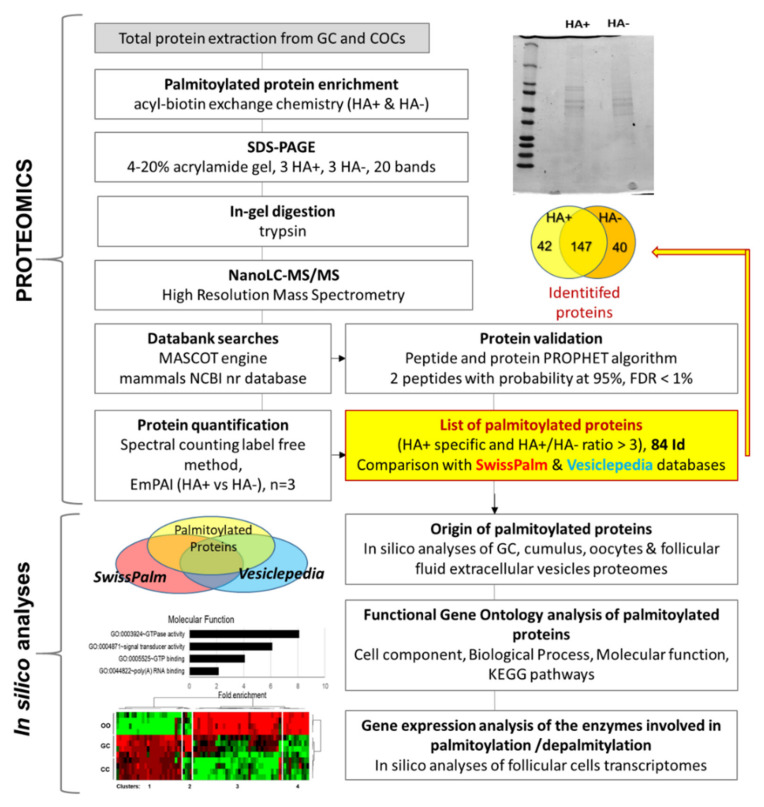
General workflow of protein palmitoylation analyses in bovine follicular cells.

In order to identify palmitoylated proteins in bovine follicular cells, we performed purification of the proteins with thioester-bonded palmitate, using an acyl-biotinyl exchange (ABE) protocol from bovine GC and COCs (workflow is shown in Appendix A, Figure A1). In total, from both palmitoyl-protein enriched hydroxylamine–treated (+HA) and non-treated (−HA) fractions, 455 proteins representing 229 unique protein clusters were identified in GC. Among them, 42 and 40 unique proteins were only identified from +HA and −HA fractions, respectively, whereas 147 GC proteins were present in both fractions. In COCs, 126 proteins regrouped in 38 clusters were identified, and of these 7 were detected exclusively in +HA fraction and 31 were represented in both +HA and −HA fractions. Proteins that were only detected in +HA fractions or significantly enriched in +HA compared to −HA (*p* < 0.05), were considered potentially palmitoylated proteins. All these potentially palmitoyl-proteins correspond to 84 proteins with unique identifiers (IDs), which are reported in Table 1. 

Five proteins (CKAP4, ITGA6, PTGFRN, S100A8, VIM) were identified from both COCs and GC samples, and six proteins were only identified from COCs: DCD, LDHC, PRR9, SELENBP1, S100A9 and TUBB4B (Table 1, shown in italics). 

This set of 84 palmitoyl-proteins was compared with a SwissPalm database containing the proteins for which S-palmitoylation was found in at least one palmitoyl-proteome in different species [3]. Analysis using *Bos taurus* SwissPalm annotated dataset (65 bovine protein IDs), revealed only 10 proteins (11.9%) common with our list of 84 potentially palmitoylated proteins (CANX, CKAP4, CD58, CTNND1, DNAJC5, GNAS, GNAI1, ITGA6, M6PR TMX3), other were for the first time reported as palmitoyl-proteins in bovine. However, when comparing with human SwissPalm dataset (4598 protein IDs), 76 out of 84 proteins were already detected in one or more human palmitoyl-proteomes (marked by “+” in Table 1), including 10 proteins reported in bovine palmitoyl-proteomes. Palmitoylation of 22 proteins was validated with two or more different methods in human (marked by “++” in Table 1). 

For six of eight follicular palmitoyl-proteins, which were not previously detected, the palmitoylation sites were predicted: CRELD1, PLSCR2, PRR9, ROR2, S100A8, STRA6. The palmitoylation of the proteins H2AC8 and TRFC were not referred in SwissPalm datasets and thus reported for the first time in this study. 

Using Membrane Proteome database (Membranome, https://membranome.org/, accessed on 22 March 2021 which holds structural and functional data of more than 6000 single-helix (bitopic) transmembrane proteins TMPs [51], we found that 32 proteins from our list were bitopic TMPs (marked “TMP” in Table 1). Among them, the palmitoylation was experimentally detected in 30 proteins and predicted in two proteins (PLSCR2 and ROR2). Moreover, 31 out of 32 palmitoylated TMPs were identified in different extracellular vesicle-proteomes, except PLSCR2 (Table 1).

**Figure 1:**
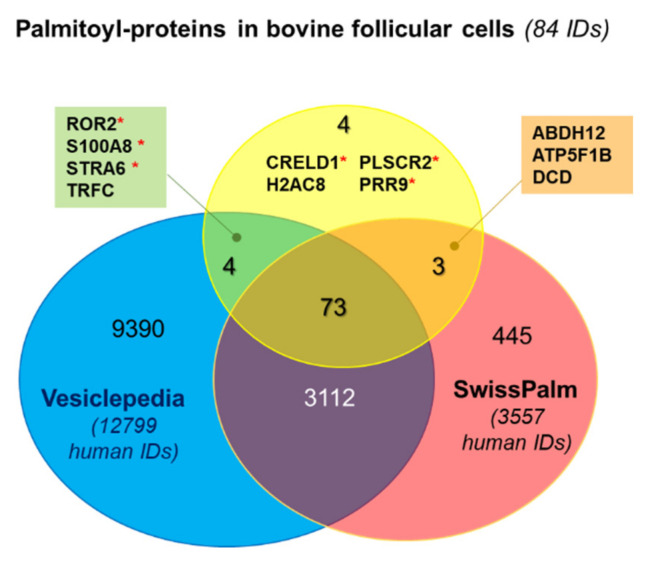
Comparative analysis of palmitoyl-proteins identified in bovine follicular cells. Venn diagram shows the comparison of the list of bovine palmitoylated proteins (84 unique IDs) with human databases SwissPalm (palmitoylated proteins, 3557 IDs) and Vesiclepedia (extracellular vesicle proteins, 12,799 IDs). Red asterisks signify that palmitoylation sites were predicted in this protein.

We have compared the list of follicular 84 palmitoyl-proteins with human EV proteomes, mainly exosomes from different biological fluids and cell secretions, as referenced in the Vesiclepedia database. Indeed, 77 out of 84 protein IDs (91.7%) were already identified as a part of human EV protein cargo (Figure 1). To compare, only 3339 out of human EV proteins (26.1%) were found palmitoylated according to the SwissPalm human dataset. 

**Figure 2:**
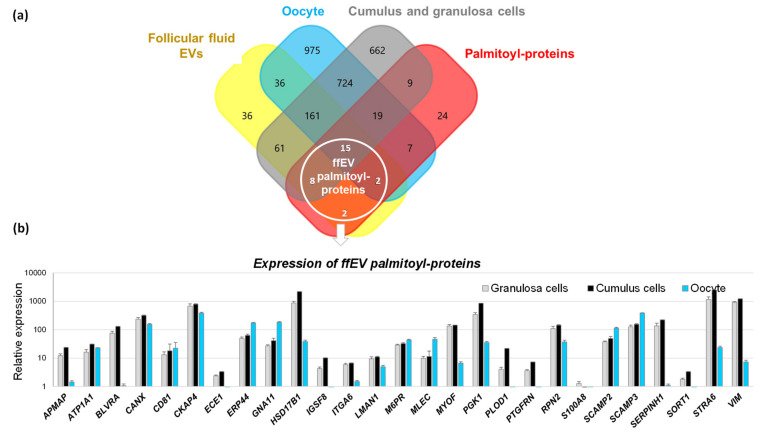
Analyses of palmitoylated proteins identified from bovine follicle. (**a**) Comparison of bovine proteomes of follicular fluid extracellular vesicles, GC [45], cumulus cells [52], and oocytes [53] with the list of palmitoylated proteins here identified from ovarian follicular cells. The 27 proteins shared with follicular fluid EV proteome are encircled. (**b**) Gene expression patterns of 27 palmitoyl-proteins detected in follicular fluid EVs by transcriptome analysis (GSE149151). Bars are mean values of 4 independent replicates ±SEM.

We then compared the lists of the proteins identified from bovine follicular fluid exosome-like EVs (294 IDs) [45], cumulus cells (1381 IDs) [52], granulosa cells (418 IDs) [45], and bovine oocytes (1936 protein IDs) [53] with the list of follicular palmitoyl-proteins (Figure 2a). 32 IDs, 48 IDs and 43 IDs of follicular palmitoy-proteins were shared with GC, CC and oocyte proteomes, respectively. 27 IDs of palmitoyl-proteins were found in follicular fluid EVs. In order to reveal possible origin of these palmitoylated EV proteins, expression of correspondent genes was analyzed in GC, CC and oocytes. As shown in Figure 2b, most of the genes were overexpressed in somatic follicular cells (GC and CC), or similarly expressed as compared to the oocytes. The data of differential analysis is reported in Supplementary Materials (Table S1).

In summary, the comparative analyses of proteomic data suggests that a significant portion of here identified palmitoylated proteins are TMBs and could be a part of protein cargo of exosome-like EVs released and/or captured by GC, CC and the oocyte. 

### 2.2. Western Blot Analysis of Palmitoyl-Proteins in Follicular Fluid Extracellular Vesicles

**Figure 3:**
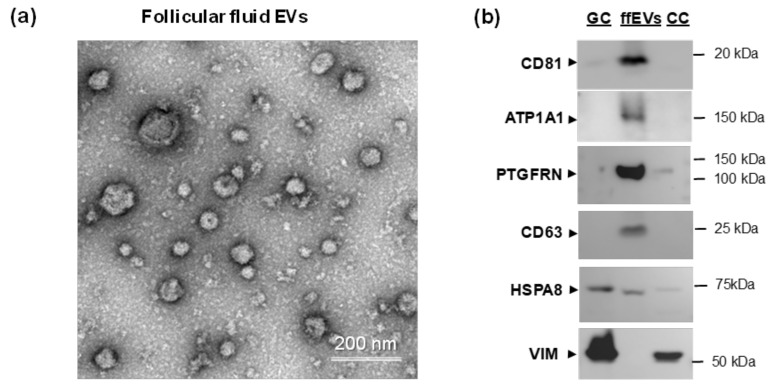
Analysis of potential palmitoyl-proteins in follicular granulosa cells (GC), cumulus cells (CC) and small extracellular vesicles extracted from follicular fluid (ffEVs). (**a**) Representative transmission electron microscopy images of ffEVs. (**b**) Western blot analysis of CD81, ATP1A1, VIM and PTGFRN in bovine GC, CC and ffEVs. Tetraspanin CD63 and heat shock protein A8 (HSPA8) were used as known EV markers [54].

To confirm the presence of potentially palmitoylated proteins in follicular fluid EVs, the small ffEVs were isolated from follicular fluid by differential centrifugation and precipitated at 100,000× *g*: (Figure 3a). Electron microscopy analysis revealed that this preparation was enriched in membrane coated exosome-like nanoparticles, which mean diameter was 60.1 ± 22.5 nm (*n* = 606). 

Western-blot analyses of total proteins from GC, CC and ffEVs were performed in order to detect several protein-candidates identified as palmitoylated in this study (Figure 3b). Proteins CD81, ATP1A1 and PTGFRN were detected enriched in ffEVs, similar to EV marker tetraspanin CD63. In contrast, EV marker HSPA8 –extracellular heat shock protein, was also abundant in GC and CC. Vimentin (VIM) was only detected in GC and CC, but not in ffEVs.

### 2.3. Gene Ontology Analyses of Palmitoylated Proteins

Enrichment analysis of cellular component GO terms of 84 palmitoyl-protein IDs is shown in Table 2. Significant enrichment was shown for the Lysosome (51 out of 84 proteins, 6-fold enrichment) and Exosomes (46 proteins, 4.4-fold enrichment). 41 proteins were associated with the Plasma membrane, particularly with Golgi apparatus (20 proteins), Endoplasmic reticulum (22 proteins) and Mitochondrion (22 proteins).

In this case, 10 proteins were matched with GO term Cytoskeleton. The most enriched GO terms in cellular components were ER-Golgi intermediate compartment, Recycling endosome membrane, and Trans-Golgi network membrane (25-fold, 54-fold and 126-fold enrichment, respectively). 

**Figure 4:**
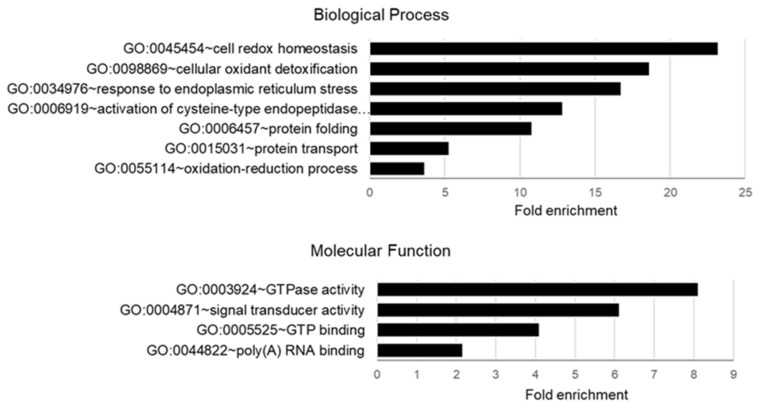
Analysis of functional GO enrichment of biological process and molecular functions in bovine palmitoylated proteins identified in follicular cells.

Analysis of Biological Process and Molecular Function GO enrichment of palmitoylated proteins from follicular cells using DAVID software is shown in Figure 4. The most enriched biological process was related to GO term Cell Redox Homeostasis, represented by the proteins TMX1, ERP44, NNT, PRDX6, AIFM1, TMX4, and TMX3. One of the he most enriched molecular function was related to GO GTPase activity, Signal Transducer Activity and GTP Binding, represented by the proteins TUFM, GNA13, RAP2A, GNAI1, GNA11, GNAS and TUBB4B.

In addition, detailed GO analysis of the list of 84 follicular palmitoyl-proteins revealed their particular association to GO term Protein Localization to Membrane, in accordance with numerous TMPs identified. Significant enrichment of palmitoyl-proteins to several GO terms related to biological processes and molecular functions was observed (false discovery rate < 0.01). Thus, GO:0055114 Oxidation-Reduction Processes regrouped the proteins HSD17B1, TMX4, TMX1, AIFL1, SELEBP1, PRDX6, FASN, PLOD1, DLAT, ACAT1, PGK1, ACAA2, ALDH6A1, LDHC, IDH2, GNAS, BLVRA and SCCPDH. Proteins S100A9, S100A8, SCAMP3, SCCPHD1, MLEC, CKAP4, DNAJC5, CD36, ERGIC3, LMAN1, ERP44, PI4K2A, CANX, TUBB4B, PRDX6, CD81, TMX3, CD58, SCAMP2, SORT1, VALP3, SCARB2 and M6PR are associated with GO:0016192 Vesicle Mediated Transport. Proteins VAMP3, SCARB2, TMX3, TMX4, TMX1, MAN1B, ERP44, AIFM1, CD58, TUBB4B, PRDX6, LMAN1, CD36, ACAT1, ACAA2, PGK1, SIGMAR1, SERPINH1, MMP14, GNAS, GN11, GNAI1, GNA13, S100A9 and S100A8 are related to GO:0006950 Response to Stress. Moreover, 40 out of 84 palmitoyl-proteins matched GO:0003824 Catalytic activity, notably Oxidoreductase activity (HSD17B1; TMX3, TMX1, AIFM1, SELEBP1, PRDX6, PGK1, PLOD1; FASN; LDHC, IDH2, NNT, BLVRA, SCCPDH, ALDH6A1), and GTPase Activity (GNA11, GNAI1, GNAS, GNA13, TUFM, TUBB4B, RAB2A). 25 palmitoyl-proteins are related to GO:0036094 Small Molecular Protein Binding, notably GO:0005102 Signalling Receptor Binding (S100A9, S100A8, GNA11, GNAI1, GNAS, GNA13, ROR2, STRA6, HSD17B1, RAPA2, OAT, ATP1A1, etc), GO:0050662 Coenzyme Binding (OAT, HSD17B1, ACAT1, AIFM1, PLOD1; FASN, ALDH6A1, IDH2, NNT), and GO:1901567 Fatty Acid Derivative Binding (CD36, S100A8, S100A9). 

**Figure 5:**
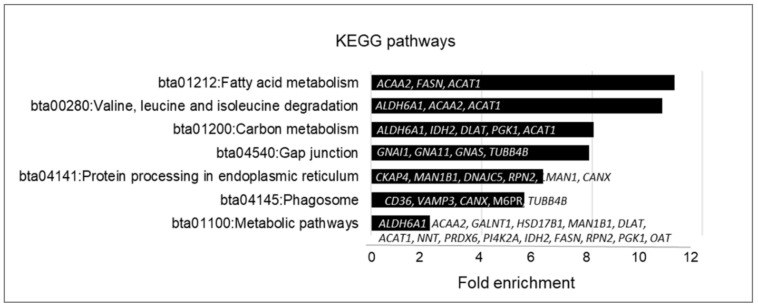
Enrichment analysis of biological pathways GO terms of the genes coding palmitoylated proteins, identified in bovine ovarian follicular cells, performed using DAVID 6.8 (https://david.ncifcrf.gov/home.jsp, accessed on 10 March 2021). Only significantly enriched KEGG pathways are shown.

Although palmitoylated proteins identified in follicular cells have many different functions, several biological pathways GO were significantly (*p* < 0.05) enriched (Figure 5). They include metabolic pathways related to Fatty Acid Metabolism (bta01212: 11-fold enrichment), Carbon Metabolism (bta01200: 8-fold enrichment), Amino Acid Degradation (bta00280: 10.5-fold enrichment), Protein Processing in ER (6.2-fold enrichment), Gap-junction (bta04540: 7.9-fold enrichment) and Phagosome (5.5-fold enrichment). 

### 2.4. Expression of Palmitoylation and Depalmitoylation Enzymes in Bovine Ovarian Follicular Cells

**Figure 6:**
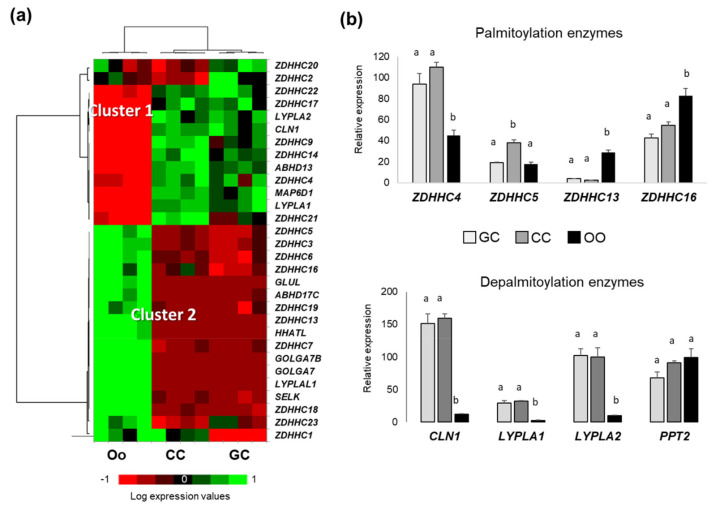
Expression patterns of the genes involved in protein palmitoylation detected by microarray hybridization. (**a**) Heat map representation of differential expression of the genes coding palmitoylation and depalmitoylating enzymes in granulosa cells (GC), cumulus cells (CC) and oocyte (OO) (*n* = 4 per each cell type). (**b**) Gene expression of the most abundant parmitoyl-transferases and acyl-thioesterases. Bars are mean values of four independent samples ±SEM. Different letters signify significant differences at *p* < 0.05.

To characterize molecular machinery of protein palmitoylation in ovarian follicular cells, expression of 31 genes included in GO:0018345 (protein palmitoylation; the covalent attachment of a palmitoyl group to a protein, and 10 genes of GO:0002084 (protein depalmitoylation; the removal of palmitoyl groups from a lipoprotein) were analyzed in bovine oocytes, follicular granulosa cells (GC), and cumulus cells (CC). From the global transcriptome dataset earlier obtained on bovine GC, CC, and oocytes (GEO accession GSE149151), we could retrieve the normalized expression values for 37 out of 41 genes coding for palmitoylating and depalmitoylating enzymes, and performed differential analysis of gene expression in GC, CC and oocyte (Supplementary Materials, Table S2). In this case, 29 genes showed differential expression (*p* < 0.05) between these cells (Figure 6), and eight genes (palmitoyl-transferases *ZDHHC8*, *ZDHHC12*, *ZDHHC15* and *ZDHHC24*, palmitoyl-protein thioesterase 2 *PPT2*, and alpha-beta hydrolases *ABHD12* and *ABHD17A*) showed similar expression level in GC, CC, and oocytes. Heatmap representation of normalized gene expression values showed that 13 differential genes overexpressed in somatic cells (GC and CC) compared to the oocytes (Figure 6a, cluster 1). Among them, there are the genes *CLN1* (coding PPT1), acyl-protein thioesterases *LYPLA1* and *LYPLA2*, and palmitoyltransferases *ZDHHC4* and ZDHHC5 (Figure 6b). Cluster 2 (Figure 6a) included the genes, which are expressed relatively higher in oocyte compared to CC and GC, as several palmitoylation enzymes including *ZDHHC13* and *ZDHHC16* (Figure 6b).

Therefore, bovine oocytes, CC and GC express different enzymes, which are involved in protein palmitoylation and depalmitoylation, and their expression patterns differ between these cell types.

### 2.5. Detection of Palmitoylation and Depalmitoylation Enzymes in Bovine Follicular Cells Proteomes

To investigate expression of palmitoylation and depalmitoylation enzymes in bovine follicular cells and oocytes, we have compared the list of 41 known enzymes with the lists of proteins identified from bovine oocytes [53], cumulus cells [52,56], mural GC, and extracellular exosome-like EVs from follicular fluid [45]. Indeed, LYPLA1 and PPT2 were found among 593 proteins identified in bovine GC [45]; PPT1, PPT2 and LYPLA2 were found among 2018 proteins identified in bovine oocytes [53], and PPT1, LYPLA1 and LYPLA2 proteins were reported in the list of 1703 identified proteins from CC surrounding immature oocytes [52]. PPT1 and LYPLA2 were also identified from CC surrounding in vivo and in vitro mature oocytes [56]. However, only one palmitoyltransferase, ZDHHC13, was identified from bovine mature oocytes [53], and from CC of in vivo mature COCs [56]. Neither palmitoylation nor depalmitoylation enzymes were detected in the list of proteins identified in follicular fluid exosome-like EVs [45]. 

Thus, in silico analysis of follicular cells transcriptomes and proteomes revealed the most abundant enzymes, which can regulate protein palmitoylation in oocytes, GC, and CC.

### 2.6. Immunofluorescence Analysis of Palmitoylation and Depalmitoylation Enzymes in Bovine Follicular Cells

**Figure 7:**
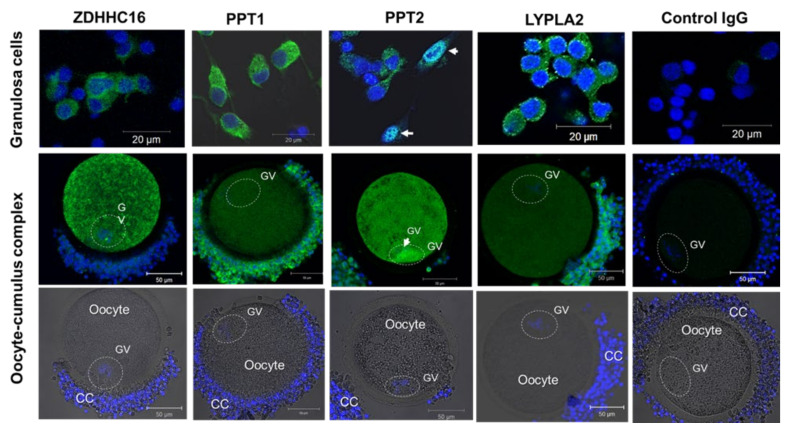
Immunofluorescence detection of protein acyl-transferase ZDHHC16, palmitoyl-protein thioesterases PPT1 (CLN1) and PPT2, and acyl-protein thioesterase LYPLA2 by confocal microscopy in bovine granulosa cells and cumulus-oocyte complexes treated with specific primary and fluorochrome-coupled secondary antibodies. For the control, rabbit and mouse IgG was used instead of primary antibodies. Specific IF labelling is shown in green. Hoechst33342 marks nuclear chromatin in blue. The position of germinal vesicle (GV) is marked with dotted line ovals. Arrows indicate nucleus with PPT2 labelling.

Using immunofluorescence (IF), we have analyzed the localization of ZDHHC16, PPT1, PPT2, and LYPLA2 in GC and cumulus-oocyte-complexes (COCs) (Figure 7). In granulosa cells, ZDHHC16, PPT1, PPT2 and LYPLA2 were localized mainly to cytoplasm, but PPT2 was also detected in the nucleus of several cells (marked by arrows). 

In COCs, ZDHCC16 demonstrated strong heterogeneous labelling within the ooplasm and, at a lower level, in the cytoplasm of attached CC. Both PPT1 and PPT2 enzymes were also detected in the oocytes and surrounding CC. PPT1 was relatively homogenously localized through the ooplasm, but more significant abundance of PPT1 was observed in CC cytoplasm. In contrast, PPT2 was more abundant in the oocyte, where it was spread through the ooplasm and more concentrated to oocyte nucleus (germinal vesicle, GV). In CC, PPT2 was observed not only in the cytoplasm by also in the nuclei. 

Therefore, intracellular localization of one potential palmitoyltransferase ZDHHC16, palmitoyl-protein thioesterases PPT1 and PPT2, and acyl-thioesterase LYPLA2 was confirmed at the protein level in bovine oocytes, CC and GC. 

### 2.7. Expression of Palmitoylation and Depalmitoylation Enzymes in Cumulus Cells and Oocytes during Oocyte Maturation

**Figure 8:**
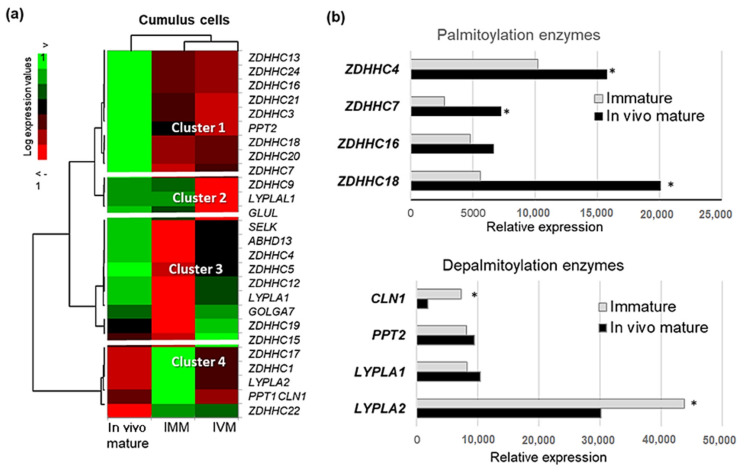
Gene expression protein palmitoylation related enzymes in bovine cumulus cells (CC) before and after oocyte maturation. (**a**) Heat map representation of gene expression in CC surrounded either immature oocytes (IMM), or in vitro mature oocytes (IVM), or in vivo mature oocytes. (**b**) Expression of the most abundant parmitoyl-transferases and acyl-thioesterases in bovine CC before and after 24 h in vitro maturation (IVM). Significant difference at *p* < 0.05 is marked by asterisks (*).

We have analyzed transcriptomic data that were previously obtained on bovine CC, which surrounded either immature oocytes, or the oocytes matured in vivo, or the oocytes after 24 h of in vitro maturation (IVM) [57]. Differential analysis of expression of the genes coding palmitoylation/depalmitoylation–related proteins in GC, CC and oocytes is reported in Appendix B (Table A1) and represented in Figure 8.

Normalized expression values are presented as a heatmap (Figure 8a). Four clusters of differentially expressed genes were determined. Cluster 1 contains nine genes overexpressed after in vivo maturation compared to immature and IVM, eight of them are ZDHHCs. Cluster 2 represents three genes down-regulated during IVM. Genes classified to cluster 3 were up-regulated during in vivo maturation and IVM, whereas cluster 4 contains mainly down-regulated genes compared to immature state. Figure 8b demonstrates relative mRNA expression in CC before and after in vivo maturation of the most abundant PATs (genes *ZDHHC4*, *ZDHHC7*, *ZDHHC16*, *ZDHHC18*), and depalmitoylation enzymes (genes *CLN1*, *PPT2*, *LYPLA1* and *LYPLA2*). Expression of *ZDHHC4*, *ZDHHC7* and *ZDHHC18* was significantly increased whereas expression of *CLN1* and *LYPLA2* significantly decreased during maturation (*p* < 0.05). Expression of many others ZDHHCs also increased in CC during oocyte maturation in vivo and/or in vitro (Appendix B, Table A1).

**Figure 9:**
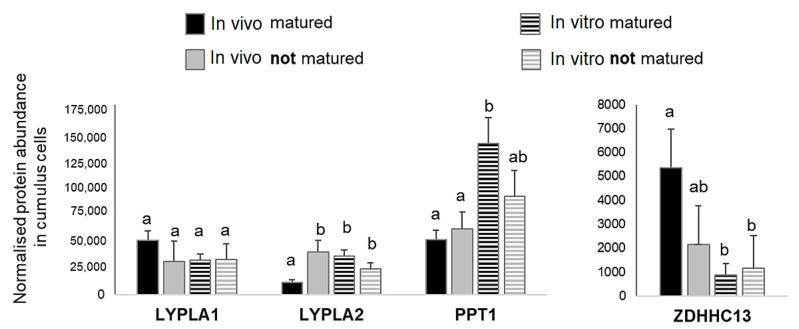
Protein abundance in bovine cumulus cells, surrounding the oocytes either matured or failed to mature after in vivo and in vivo maturation. The values of normalized protein abundance in individual bovine cumulus (*n* = 5 per condition) from the recent proteomic study [56] were compared using ANOVA and Tuckey post-hoc test. Different letters signify significant difference at *p* < 0.05.

At the protein level, analysis of the bovine CC proteome from in vivo or in vitro matured COCs [56] demonstrated that PPT1 and LYPLA2 enzymes were about 2.5-fold less abundant in CC of the oocytes matured in vivo than in vitro (*p* < 0.05); in contrast, ZDHHC13, the only one PAT detected in CC, was significantly more abundant in in vivo matured CC compared to in vitro matured (Figure 9). In addition, LYPLA2 was significantly up-regulated in CC of the oocytes that failed to mature in vivo comparing to fully matured (*p* < 0.05); other enzymes did not show any significant difference. 

By analyzing quantitative proteomics data on bovine immature and in vitro mature oocytes from the recent study [53], we found that PPT1 and LYPLA2 were significantly more abundant in mature oocytes than in immature ones (6.3-fold and 3.6-fold, respectively). In contrast, PPT2 was identified from only immature oocytes. 

## 3. Discussion

### 3.1. Palmitoylated Proteins and Their Role in Ovarian Follicular Cells

In the present study, we have isolated and identified for the first time 84 potentially palmitoylated proteins from ovarian follicular cells in bovines. According to recent data presented in SwissPalm, 19.86% of human proteins were detected or predicted to be palmitoylated; however only 0.54% of *Bos taurus* proteins were identified as palmitoyl-proteins [3]. This is certainly due to few studies of palmitoyl-proteomes analyses in bovine cells reported until now. The palmitoyl-proteome of bovine ocular lens fiber cells was obtained using a similar ABE approach and revealed 174 potentially palmitoylated proteins [19]; other studies in cattle reported palmitoylation of several candidate proteins: G protein-coupled receptor rhodopsin [58], retina pigment epithelium protein RPE65 [59], or pulmonary surfactant proteins B and C [60]. Taking in account relatively rare biological material as GC and COCs, and numerous purification steps to obtain +HA fractions, only the most abundant proteins could be identified. Among them, 76 proteins (90.5%) were already identified in a palmitoylated form in human cells [3], and 28 follicular palmitoyl-proteins were already detected as palmitoylated in bovine lens fiber cells [19]. Therefore, eight palmitoyl-proteins were reported here for the first time: CRELD1, H2AC8, PLSCR2, PRR9, ROR2, S100A8, S1TRA6 and TRFC. Potential palmitoylation sites were predicted for six of them; two other proteins were either specific (TRFC) or significantly enriched in +HA fractions (H2AC8, fold change 11), and therefore may be considered as novel potential palmitoyl-proteins. Reversibility of protein palmitoylation may explain the presence of the same proteins in both +HA and −HA fractions, as the protein molecules may be either palmitoylated or not in the same cells. Many proteins identified as palmitoylated in bovine follicular cells were reported in proteomes of bovine oocytes, CC and follicular fluid EVs; however, the role of palmitoylation in functioning of each of these proteins in situ remains to be investigated.

The identified follicular palmitoyl-proteins are involved in different molecular functions and biological processes, similar to other palmitoyl-proteomes. Functions of reported here palmitoyl-proteins were related to protein processing and secretion, binding of small molecules as ions, nucleotides or lipids, protein-protein binding, FA transport, mitochondrial activity and energy metabolism, various enzymatic activities, signal transduction and oxidative stress response. Many of them are transmembrane proteins, participating in signal transduction in response to hormonal stimulation through different pathways, such as PI3K signaling (S100A9), MAPK cascades (G-proteins), EGF-signaling (SIGMAR1, CREDL1), IGF1-signalling (ITGA6) or WNT-signaling (CTNND1, ROR2) and others. 

Lipid and carbohydrate metabolism were among the most enriched functional pathway of identified palmitoyl-proteins. According to Swisspalm database, many proteins involved in FA metabolism, including FA transporters, enzymes of lipid synthesis and transformation are palmitoylated in human cells. In our study, CD36, a major lipid transporter of FAs to the cell, was identified in follicular cells in palmitoylated form. In mice adipocytes, only palmitoylated CD36 mediates FA uptake through caveolar endocytosis, and depalmitoyalation of CD36 initiates the formation of endocytic vesicles, which assure delivery of FAs inside the cell and target them to lipid droplets [61]. Therefore, palmitoylation of FA transporters and other lipid metabolism related proteins as FASN, ACAA2, HSD17B1 and other, may be involved in the mechanisms of FA uptake and lipid metabolism in ovarian cells, which are different in follicular cells and oocyte [62]. 

Gap-junction functional pathway and vesicle mediated transport were among the enriched GO terms of follicular palmitoyl-proteins. These functional activities and pathways largely imply bidirectional oocyte-somatic cells communications and are crucial for oocyte growth and maturation within the follicle. Cell interactions permanently occur between the oocyte and surrounding cells through cell-to-cell communications via gap-junctions [48,63,64,65] and extracellular vesicles [47]. Indeed, the most of the proteins detected as palmitoylated in this study were also part of protein cargo of EVs of different origin according to EV-proteome database [66].

Protein palmitoylation has a role in targeting of the proteins not only to plasma membranes but also to the nucleus, spindle and cleavage furrow [67], which changed their structure during oocyte meiotic divisions. In mammals, oocyte meiotic maturation is accompanied by cytoplasmic maturation, which includes great ultrastructural changes not only to nuclear DNA, which carry out chromosome duplication, meiotic resumption and first polar body extrusion, but also to Golgi, mitochondria, vesicular bodies, cortical granule, and lipid droplets within the oocyte [68,69]. In bovines, oocytes are transcriptionally silent during maturation; the proteins are synthetized from maternal mRNA storage [70] and undergo different post-translational modifications. Although protein phosphorylation is the most important modification present in 30–50% of detected proteins [71], protein palmitoylation also occurs in follicular cells. In the present study we showed expression of numerous PATs and depalmitoylation enzymes and identified a set of palmitoyl-proteins in follicular cells, several of which are known to be associated with oocyte maturation. According to actual model of meiosis in mammals, oocyte meiotic arrest is maintained by a constitutively active GPCRs GPR3, or GPR12 through activating heteromeric G-protein Gs alpha (GNAS), which stimulates cAMP-dependent pathway leading to high intracellular levels of cAMP and cGMP [20,72]. Palmitoylation of specific proteins such as Ras [23] or Gs alpha [24] is required to activate or block meiosis in Xenopus oocytes. In this study, several palmitoylated G-protein subunits (GNAS, GNAI1, GNA11, GNA13) and Ras-related protein (RAP2A) were detected in bovine follicular cells and oocyte. In human cells, palmitoylation of Cys residues on the intracellular side of GPCRs, controlling their desensitization and internalization, is required for the capacity of GPCRs to interact with membrane and to bind ligands for exerting their functions [11]. Bovine oocytes express genes of numerous GPCRs, including GPR3, GPR12, and GNAS, which are involved in meiotic arrest in mice, human and pig [72]. Thus, these G-proteins and GPCRs are likely palmitoylated in bovine oocyte and participate in signaling that triggers oocyte meiosis resumption. 

### 3.2. Palmitoylation Protein Sorting and Involvement in Communications between Follicular Cells

Most of the palmitoyl-proteins identified here were matched to different membrane compartments: Golgi, recycling endosome membrane, lysosomes, exosomes and other membrane structures. This localization was expected, because the major functions of palmitoylation are stable membrane anchoring of soluble proteins and regulation of protein sorting by either partitioning of proteins into cholesterol-rich lipid rafts, or modulating protein-protein interactions, or regulation of ubiquitination status [73]. In fact, palmitoylation is involved in either retention or anterograde trafficking of proteins at the ER–Golgi or protein cycling within the endosomal/lysosomal system. Protein sorting from Golgi to the endosomal/lysosomal compartments is mediated by three trans-membranes receptors: sortilin (SORT) and two mannose 6-phosphate receptors. In human cells, palmitoylation of these receptors served to shift their fate from degradation to recycling and prevent their degradation [74]. In our study, we have identified two palmitoylated receptors: SORT and cationic-dependent 6-phosphate receptor M6PR, which could be implicated in trafficking of the proteins to the lysosomal compartment in follicular cells. Lysosomes contain soluble enzymes for the degradation of proteins, lipids, and cytoplasmic organelles, and PPT1 is one of 60 lysosomal hydrolases, which depalmitoylates proteins prior to their degradation. Palmitoylation therefore prevents the proteins from degradation and could target their sorting to exosomes, small EVs of endosomal origin [38,75]. Recent study of the global palmitoyl-proteome of EVs released from prostate cancer cells demonstrated that inhibition of palmitoylation reduced the abundance of several palmitoyl-proteins in small EVs [76]. 27 proteins detected as palmitoylated in this study were earlier identified in bovine follicular fluid exosome-like EVs [45], and likely originated from different types of cells. We demonstrated by Western blot the enrichment of several of them, including ATP1A, PTGFRN, and one of the main markers of EVs, tetraspanin CD81, in ffEVs. Other palmitoyl-proteins potentially could be also present in ffEVs, because 91.7% of reported here proteins were already identified from EVs released by human cells. Thus, protein palmitoylation seems be important for protein sorting from the cells to ffEVs and their transport to target cells through follicular fluid. This was particularly applied for palmitoylated transmembrane proteins, which were all except one, detected in bovine ffEVs [45]. Within the follicle, follicular fluid EV-mediated transport of different active proteins plays crucial role in communication between the oocyte and the surrounding follicular cells and mediates the response of the recipient cells to different stress stimuli [47]. Different proteins involved in stress response and here identified as palmitoyl-proteins are present in ffEVs and could be involved in regulation mechanisms of follicular development and oocyte maturation. Protein palmitoylation may therefore represent an important molecular mechanism involved in the regulation of trafficking of related proteins between the cells and in the mediation of the signaling inside the follicle through ffEVs exchanges. 

### 3.3. Regulation of Protein Palmitoylation within Ovarian Follicle 

Regulation of protein palmitoylation within the follicle may affect not only sorting, localization and activity of the modified proteins, but may also regulate stress response to lipotoxicity, which occurs when level of free saturated FAs, especially palmitate, is elevated and affects oocyte quality [76,77,78]. An excess of palmitate induced ER stress likely through defects in, or dysregulation of protein palmitoylation in human cells [79,80]. Partial inhibition of protein palmitoylation by 2-bromopalmitate attenuated caspase activation, decrease of insulin secretion and cell death in response to excess of free palmitate in pancreas beta cells [79], and increased mTORC1 activation and lipotoxicity protection in hepatocytes in vitro [80]. 

The tight relationship between protein palmitoylation and intracellualr lipid metabolism metabolism is also supported by high depalmitoylation activity in the mitochondria of mammalian cells, suggesting a role of palmitoylation in mitochondrial lipid homeostasis, and therefore cell functioning and viability [81]. 

In the present study, we characterized enzymatic machinery of protein palmitoylation in bovine ovarian GC, CC and enclosed oocytes, for the first time. According to transcriptomic data, most of the genes coding the enzymes, which either catalyze protein S-palmitoylation (PATs and associate proteins), or remove palmitate (APTs and associate proteins) demonstrated cell-specific expression pattern of protein palmitoylation in follicular cells and enclosed oocyte. In human, substrate-specific PAT activity was reported for 17 out of 23 known ZDHHC proteins [31]. According to our analysis, at least 12 out of 23 ZDHHCs expressed in GC, CC and oocyte. From these, transcripts of ZDHHC3, 4, 5, 6, 13, 14 and 16 was the most abundant. Genes *ZDHHC4* and *ZDHHC14* were overexpressed in somatic follicular cells (GC and CC) in contrast to *ZDHHC3*, *ZDHHC5*, *ZDHHC13* and *ZDHHC16*, which demonstrated higher expression in the oocytes. In concordance, although ZDHHC16 protein was detected by immunofluorescence in follicular somatic cells, the most intensive immunostaining was observed in the oocyte. Protein palmitoylation occurs mainly at the plasma membrane and at the Golgi apparatus, although palmitoylated proteins could be localized to various subcellular organelles, including mitochondria, nucleus, ER, and others (Fukata et al., 2016). Granular aspect of ZDHHC16 immunofluorescence labeling corroborates with preferential location of several PATs to Golgi, ER and plasma membranes [12]. 

A protein can be palmitoylated specifically by single ZDHHC, or by several PATs. In mice, FA transporter CD36 is specifically palmitoylated by ZDHHC5 [61]. ZDHHC7 and ZDHHC21 are conserved specific PATs for endogenous palmitoylation of estrogen, progesterone, and androgen receptors, their membrane trafficking and signal transduction, as was shown in cancer cells [82]. In *Xenopus* egg, ZDHHC3 palmitoylates a protein Gs alpha (GNAS), and oocyte-specific knockdown of ZDHHC3 led to the acceleration of progesterone-induced meiosis resumption, demonstrating the importance of PAT-mediated palmitoylation in oocyte meiosis [24]. Transcriptomic analysis of bovine CC showed that the most of detected ZDHHCs (ZDHHC3, 4, 5, 7, 12, 13, 16, 18, 20, 24) increased their mRNA expression during oocyte maturation in vivo and/or in vitro, consolidating their possible role in CC to regulate oocyte meiosis. Detection of ZDHHC13 in the proteomes of only mature and not immature oocytes [53] and in CC from mature oocytes [52] indicates up-regulation of this enzyme in COCs during oocyte maturation at protein level, and therefore suggests its possible involvement in meiosis. Recently, 254 potential substrates of ZDHHC13 were identified using *Zdhhc13*-deficient mice model [83]. Among these, proteins of mitochondrial dysfunction and lipid metabolism were over-represented, and several of these proteins were also detected palmitoylated in bovine follicular cells in this study. Thus, catenin delta 1 (CTNND1) was confirmed to be palmitoylated by ZDHHC13 in mice liver [83], and in our study, CTNND1 was also identified as palmitoyl-protein, moreover it was detected in bovine oocyte proteome [53]. Proteins ACAA2, CD81, FASN and SELENBP1 were found as potential substrates of ZDHHC13 in mice, and also they were identified palmitoylated in follicular cells in our study; in addition, these proteins were also present in bovine oocytes [53]. Thus, palmitoyltransferase ZDHHC13 and its potential substrates are present in bovine oocytes, moreover, ZDHHC13 was more abundant in mature oocyte and CC than in immature state, that could imply the importance of this PAT for oocyte maturation in cattle.

Protein depalmitoylation is promoted by specific enzymes including APTs, PPTs and ABHD-containing proteins [35]. In our study, we demonstrated that genes of APTs *LYPLA1* and *LYPLA2*, palmitoyl-thioesterases PPT1 (gene *CLN1*) and PPT2 (*PPT2)*, as well as two ABHD enzymes (genes *ABHD12* and *ABDH17C*) strongly and cell-specifically expressed in follicular somatic cells and enclosed oocyte. *CLN1* and *ABHD12* significantly more expressed in GC and CC, whereas *PPT2* and *ABDH17C* overexpressed in the oocytes. According to proteome analysis of bovine granulosa cells [45], cumulus cells [52,56] and oocytes [52,53], the proteins LYPLA1, LYPLA2, PPT1 and PPT2 were relatively abundant in follicular cells. In addition, intracellular localization of PPT1, PPT2 and LYPLA2 in GC and COCs was here confirmed by immunofluorescence analyses and corresponds to known intracellular locations of these enzymes. Morphological aspects of LYPLA2, PPT1 and PPT2 immunofluorescence suggests that they likely positioned to plasma membranes and vesicular structures in the cytoplasm. Such labeling corroborates with preferential location of PPT1 to lysosomes and APTs dispersed through cytosol, allowing protein depalmitoylation throughout the cell [73]. The only protein showing both cytoplasm and nuclear labeling was PPT2, which has palmitoy-CoA hydrolase activity but possesses distinct substrate specificity compared to PPT1 [84]. 

In CC, genes *CLN1* (coding for PPT1) and *LYPLA2* decreased during maturation of enclosed oocyte. In concordance, proteomic data revealed significantly lower abundance of PPT1 and LYPLA2 proteins in bovine CC surrounding in vitro matured oocytes compared to in vivo maturation [56]. In the oocyte, PPT1 and LYPLA2 proteins were several-fold more abundant in mature oocytes than in immature oocytes [53]. These data suggested that protein depalmitoylation is important step for oocyte meiosis, and this process is regulated differently in the oocytes and surrounding CC. Indeed, in frog oocytes, inhibition of protein palmitoylation accelerated the first steps of meiosis resumption [24]. Therefore, increase of protein depalmitoylation in the oocyte may play a role in kinetics of oocyte meiosis. 

Globally, expression modulations of palmitoylation/depalmitoylation enzymes in CC accompanied oocyte maturation, and in vivo changes of protein palmitoylation machinery were more significant than after IVM. Indeed, oocyte competence to develop embryo was higher after in vivo oocyte maturation compared to IVM in cow [85,86,87], therefore, it might be hypothesized that protein palmitoylation in COCs could be modulated by in vitro culture and consequently affected oocyte quality. 

In conclusion, it the present study we characterized molecular machinery of protein palmitoylation in bovine granulosa cells, cumulus cells and oocytes, and identified the most abundant palmitoyl-proteins from these cells. Palmitoylation is likely mediated by several highly expressed ZDHHCs, which increase their expression in CC during oocyte maturation, whereas expression of depalmitoylation enzymes decreased. In the oocyte, depalmitoylation seems to increase and thus may be involved in meiosis regulation. Follicular palmitoylated proteins are involved in different cell metabolism processes and signaling and can be transported by follicular fluid extracellular vesicles. However, the role of palmitoylation in functioning of each of these proteins in follicular cells or oocytes remains to be investigated.

## 4. Materials and Methods

### 4.1. Ethics

No experiments with living animals were performed. 

### 4.2. Chemicals

All chemicals were provided by Sigma-Aldrich (Saint-Quentin Fallavier, France) unless others stated. 

### 4.3. Recovery of Follicular Cells and Oocytes

Bovine ovaries were collected at a commercial slaughterhouse. Bovine follicular cells, mainly granulosa (GC) and cumulus-oocyte complexes (COCs) were retrieved from the antral follicles 3–8 mm in diameter by vacuum aspiration. Immature COCs with compact cumulus layers were selected and washed in TCM199-HEPES medium complemented with 0.04% BSA, and 25 μg/mL gentamycin. 

For proteomic studies, GC from the sediments of follicular fluid and TCM199-HEPES after COCs’ retrieval were centrifuged at 300× *g* for 10 min and washed by pipetting with modified McCoy’s 5A serum-free medium containing 3 mM L-glutamine and 20 mM HEPES (pH-7.6). Suspended GC were dropped off on a Percoll density medium (50% Percoll, 50% McCoy’s medium) and purified GC were collected after centrifugation (300× *g*, 30 min). Selected COCs and GC samples were then washed twice in sterile PBS, centrifuged again and the pellets kept at −80 °C until protein isolation and proteome analysis. 

### 4.4. Analysis of Protein Palmitoylation

General workflow of analyses of protein palmitoylation in bovine follicular cells included proteomic approach to purify and identify palmitoyl-proteins and in silico analyses of their functions (Appendix A, Figure A1). In addition, gene expression analysis of palmitoylation/depalmitoylation enzymes was performed. 

#### 4.4.1. Isolation and Identification of Palmitoyl-Proteins

Acyl-biotinyl exchange chemistry (ABE) technology [15] was used to purify the palmitoyl-proteins, according to the protocol described by Wan et al. [16], which we have applied with minor modifications to bovine follicular cell proteins. Detailed protocol is reported in Supplementary Materials, protocol “ABE isolation and identification of palmitoyl-proteins”. 

Briefly, total proteins were extracted from frozen pellets of GC (total amount is about 0.5 g), and COCs (*n* = 500), collected from about 120 ovaries in tree experiments, using 10 mM N-ethylmaleimide (NEM) to block the free thiols. After removing the NEM, the subsequent treatments with hydroxylamine (HA) were performed in order to cleave the palmitoylation thioester linkages. Following treatment with HPDP-biotin biotinylated free thiols. Proteins were precipitated three times using chloroform-methanol, resuspended in 4% SDS and divided into two equal portions (+HA sample and −HA sample as control). The +HA sample was diluted five-fold by addition hydroxylamine-containing +HA buffer (0.7 M HA, 1 mM HPDP–biotin, 0.2% Triton X-100, 1 mM PMSF, 1x PI pH 7.4); for the −HA samples, −HA buffer (50 mM Tris, 1 mM HPDP–biotin, 0.2% Triton X-100, 1 mM PMSF, 1x PI, pH-7.4) was added. The +HA and −HA samples were incubated at room temperature for 1 h with end-over-end rotation, and then precipitated to remove chemicals. After serial precipitations, affinity purification of biotinylated proteins was performed per each −HA and +HA sample, then precipitated using 100% trichloroacetic acid (TCA), and stored −20 °C.

For identification of palmitoylated proteins, each pellet from +HA and −HA samples was resuspended in SDS-PAGE sample buffer containing 4% SDS, 5% beta-mercaptoethanol, 125 mM Tris-HCl pH 6.8, 20% glycerol and bromophenol blue as the dye, and incubated for 5 min at 95  °C, then centrifuged at 10,000× *g* for 5 min, and SDS-PAGE was performed. For exhaustive identification, each line of −HA and +HA samples were sectioned into 20 slices which then used for in-gel digestion and nano-LC-MS/MS analysis performed using a LTQ Orbitrap Velos mass spectrometer (Thermo Fisher Scientific, Bremen, Germany) coupled to an Ultimate^®^ 3000 RSLC Ultra High Pressure Liquid Chromatographer (Dionex, Amsterdam, The Netherlands). 

For protein identification, mass spectrum ion searches were performed using Mascot search engine v2.3.2 (Matrix Science, London, UK) via Proteome Discoverer 2.1 software (ThermoFisher Scientific, Bremen, Germany). Research was performed against the mammalian non-redundant NCBI database (released July 2018). Peptides and proteins identified by MASCOT were subjected to Scaffold v4.8.4 software (Proteome Software Inc., Portland, OR, USA). Peptide and protein identifications were accepted if they could be established at greater than 95% probability by the Peptide Prophet algorithms [88,89], respectively. Proteins sharing significant peptide evidence were grouped into clusters. For each protein identified to be non-bovine, a manual blast analysis against *Bos taurus* protein databases was performed to obtain a species-specific identification of the protein. The abundance of identified proteins within one sample was estimated by calculating the Exponentially Modified Protein Abundance Index [90]. 

To characterize enriched palmitoylated proteins, +HA and −HA conditions analyzed in triplicate (R1, R2, R3), with three technical replicates per band were compared using Scaffold Q+ software (v4.4, Proteome Software, Portland, OR, USA). Label-free quantitative approach was employed. Protein clusters were defined as enriched palmitoylated proteins if they met the following conditions: (1) detection once among the three replicates only in +HA and never in control condition −HA; (2) detection in both conditions but with an enrichment in +HA with a fold change > 3. The mass spectrometry proteomics data have been deposited to the ProteomeXchange Consortium via the PRIDE [91] partner repository with the dataset identifiers PXD020540 and PXD020547. 

#### 4.4.2. Gene Expression Analysis

Microarray expression data (public repository Gene Expression Omnibus (GEO) accession number GSE149151) were used for analysis of gene expression in GC, CC and oocytes. Normalized expression values were retrieved for the genes coding the enzymes involved in palmitoylation (GO:0018345) and depalmitoylation (GO:0018345) and identified palmitoylated follicular proteins. The analysis of differential expression between the follicular cells (GC, CC and oocytes) was performed on normalized expression values of four biological replicates using XLSTAT software (Addinsoft, Paris, France) by applying ANOVA with Benjamini- Hochberg correction of *p*-values. Multiple pair analysis was performed by Tukey test. Difference at *p* < 0.05 was considered significant. Microarray expression data were validated by real time PCR for 10 genes involved in lipid metabolism in our previous study [62]; in addition, expression of several additional genes including CLN1 and PPT2 were analyzed in GC, CC and oocytes (Supplementary Figure S1), using specific primers (Supplementary Materials Table S3), as described [62].

#### 4.4.3. Functional Annotation Bioinformatics Resources

For functional annotation of palmitoyl-proteins, we have used SwissPalm database, released 3, 8 September 2019 (https://swisspalm.org/, accessed on 12 March 2021), which contains the proteins with experimentally detected palmitoylation [3], Vesiclepedia database v4.1 (http://microvesicles.org/index.html, accessed on August 2018), which contains the information about molecular composition of extracellular vesicles in different species [66,92], and Membrane Proteome (Membranome) database (https://membranome.org/, accessed on 22 March 2021) holding structural and functional data of more than 6000 single-helix (bitopic) transmembrane proteins [51].

Gene Ontology analyses were performed with FunRich software v3.1.3. [55], STRING version 11 (https://string-db.org/, accessed on 17 April 2020) and DAVID functional Annotation Bioinformatics microarray analysis (https://david.ncifcrf.gov/, accessed on 10 March 2021). 

### 4.5. Isolation of Extracellular Vesicles from Follicular Fluid

Small extracellular vesicles (exosome-like ffEVs) were isolated from the 3 pools of follicular fluid aspirated from 3–8 mm follicles by differential centrifugation, as previously described [43]. Briefly, samples of follicular fluids were clarified from cells, cell debris and apoptotic bodies by the 1st centrifugation at 300× *g* 15 min, and the second one at 2000× *g* 15 min at room temperature. The samples of clarified follicular fluid were then centrifuged 30 min at 12,000× *g* at 4 °C to remove the large EVs. To sediment the small ffEVs, supernatants were ultra-centrifuged at 100,000× *g* for 90 min at 4 °C, and the pellets were washed with 4 mL PBS and centrifuged for 90 min at 100,000× *g*. Pellets were resuspended in 25 µL of PBS per 1 mL of original follicular fluid. The preparations of ffEVs were kept at −80 °C. 

### 4.6. Transmission Electron Microscopy (TEM)

Of each fresh preparation of ffEVs, 3 µL were fixed with 3 µL of 2% glutaraldehyde solution in PBS at room temperature. TEM was performed with 3 µL of fixed EVs as described earlier [45]. Micrographs were obtained using a Hitachi HT 7700 Elexience and a JEM 1011 TEM (JEOL, Japan) electron microscopes with digital camera. Images were used for measuring EV sizes with Fiji software [93].

### 4.7. Total Protein Extraction and Western Blot

Total protein samples of GC, CC and ffEVs were prepared as described elsewhere [45]. Briefly, lysis buffer containing Tris-saline-EGTA buffer (pH-7.5) supplemented with 2 mM sodium othovanadate and 1 µg/mL of protease inhibitor cocktail (Sigma) was added to the pellets of GC, CC or ffEVs, and thoroughly mixed by pipetting. The homogenates were incubated on ice for 30 min. Then Laemmli reducing buffer containing 1% SDS, 68mM Tris-HCl, 10% glycerol and 80 mM dithiothreitol at final concentration was added to the samples. Before loading, protein extracts were heated at 99 °C for 8 min and centrifuged 5 min at 12,000× *g*. Protein samples were resolved on ready 4–12% SDS-PAGE gels (Life technologies; Saint-Aubin, France) in replicates and transferred onto nitrocellulose membrane (Pall Corporation, VWR International; France). After blocking in 5% dry milk/Tris-buffered saline/0.1%Tween (TBST), membranes were incubated with primary antibodies raised against human ATP1A1 (1/500 final dilution), CD63 (1/500 dilution), CD81 (1/1000 dilution), PTGFRN (1/500), VIM (1/1000 dilution) and HSPA8 (1/1000) at 4 °C overnight. Membranes were washed four times using TBST and incubated with corresponding secondary horseradish peroxidase (HRP)-conjugated secondary antibodies (1: 5000 to 1: 10,000 final dilution) in TBST for 1 h at room temperature. Specific signals were revealed by chemiluminescent reagent SuperSignal™ West Dura Extended Duration Substrate (Thermo-Fisher Scientific, Courtaboeuf, France). Each membrane was used to reveal at least different size proteins, after stripping of previous antibody. Signals were captured using a GeneGnome camera (Syngene; Cambridge, UK) and Genesys 1.5.4 software (Syngene). 

### 4.8. In Vitro Culture of Granulosa Cells

GC, collected and purified as described above, were diluted McCoy culture medium supplemented with 5% of foetus calf serum at a concentration 1 million cells per mL and incubated on an 8-well chamber slide (Lab-Tek^®^ Nunc, Thermo-Fisher Scientific, Courtaboeuf, France) for 24 h at 38.8 °C, in humidified thermostat at 5% CO_2_ and 20% O_2_. Culture media was removed, GC rinsed with PBS and used for immunofluorescence analysis (*n* = 3).

### 4.9. Immunofluorescence

Immature COCs (120 COCs, in three experiments) partially stripped from their cumulus and GC after 24 h of culture were washed in Tris-buffer saline (TBS), fixed in 4 in PBS containing 4% paraformaldehyde (PAF) for 20 min and then washed in TBS-0.1% BSA for 3 min at room temperature. After blocking in TBS containing inactivated sheep serum (5%) and 0.1% Triton X100 for 30 min (GC), or 1 h (COCs) at room temperature, cells were incubated overnight at 4 °C in TBS containing BSA (0.2%) with primary rabbit antibodies against human PPT2 or ZDHHC16, or LYPLA2, or mouse antibodies against human PPT1 all at 1:100 dilution. Control cells were incubated with similar concentration of mouse or rabbit was IgG (negative control). Three 5-min washes (GC), or four 30-min washes (COCs) in TBS containing 0.1% BSA and 0.1% Tween 20 were performed, then samples were incubated with 1:400 dilution of secondary Alexa Fluor 488-conjugated goat anti-rabbit IgG and/or Alexa Fluor 594-conjugated goat anti-mouse IgG (Life Technologies, Saint Aubin, France), in TBS containing 0.2% BSA for 1 h (GC), or 2 h (COCs) at room temperature. Then, four 10 min washes in TBS containing BSA (0.1%) and incubated in TBS with 2.5 µg/mL Hoechst 33258 for 15 min at room temperature to stain nuclear chromatin. Sections were then mounted using Moviol^®^ and fluorescence was observed under a Zeiss confocal microscope LSM700 (Carl Zeiss Microscopy GmbH, Munich, Germany) using 20× objective or an oil 40× objective with a numerical aperture of 1.3 and the appropriate filters. Images were captured using Zen 2012 software (black edition v8.0, Carl Zeiss Microscopy GmbH).

## 5. Conclusions

Ovarian follicular cells express a number of specific enzymes controlling protein palmitoylation and depalmitoylation, and their expression patterns are cell-specific. In bovine oocytes and surrounding cumulus and granulosa cells, reversible palmitoyation is mediated by different ZDHHCs and APTs, which are strongly expressed in these cells and varied their expression during oocyte maturation. The most abundant palmitoyl-proteins within the follicle were identified. They matched to different biological processes and pathways attributed to palmitoyl-proteins in other cells, and were enriched in the proteins regulating energy metabolism, cell signaling and extracellular transport. Palmitoylated proteins could be transported by follicular fluid extracellular vesicles and therefore might be intermediators of intra-follicular signaling pathways, involved in follicular growth and oocyte maturation. Protein palmitoylation seems to be crucial for progression of oocyte maturation in vivo and in vitro although the roles of palmitoylation for each protein involved in these processes remain to be investigated. 

## Figures and Tables

**Table 1:** List of palmitoylated proteins identified from bovine follicular cells.

ID	Protein Name	Palmitoylation (Human)	EVs Cargo	Membranome	+HA Enrichment Fold
ABHD12	abhydrolase domain containing 12. lysophospholipase	+			56
ACAA2	3-ketoacyl-CoA thiolase. mitochondrial (acetyl-CoA acyltransferase 2)	+	yes		only +HA
ACAT1	acetyl-CoA acetyltransferase 1	+	yes		3.9
AIFM1	apoptosis inducing factor mitochondria associated 1	+	yes		2.2
ALDH6A1	aldehyde dehydrogenase 6 family member A1	+	yes		only +HA
APMAP	adipocyte plasma membrane associated protein	+		TMP	3.2
ATP1A1	sodium/potassium-transporting ATPase subunit alpha-1	+	yes		2.1
ATP5F1B	ATP synthase F1 subunit beta	+			3.7
B3GNTL1	UDP-GlcNAc:betaGal beta-1.3-N-acetylglucosaminyltransferase-like protein 1 (alias B3GNT8)	+	yes	TTMP	only +HA
BLVRA	biliverdin reductase A	+	yes		only +HA
CANX	calnexin	++	yes	TMP	42
CD36	platelet glycoprotein 4 (CD36 antigen)	+	yes		only +HA
CD58	lymphocyte function-associated antigen 3	++	yes	TMP	only +HA
CD81	CD81 antigen (target of antiproliferative antibody 1)	++	yes		only +HA
*CKAP4*	*cytoskeleton associated protein 4*	++	yes	TMP	21
CLGN	calmegin	+	yes	TMP	only +HA
CRELD1	cysteine rich with EGF like domains 1	predict			4.4
CTNND1	catenin delta 1	++	yes		only +HA
*DCD*	*dermcidin isoform 1 preproprotein*	+			*6.01*
DLAT	dihydrolipoamide S-acetyltransferase	+	yes		only +HA
DNAJC5	DnaJ heat shock protein family (Hsp40) member C5	++	yes		only +HA
ECE1	endothelin converting enzyme 1	++	yes	TMP	only +HA
EPHX1	epoxide hydrolase 1	+	yes	TMP	7.2
ERGIC3	endoplasmic reticulum-Golgi intermediate compartment protein 3	++	yes	TMP	only +HA
ERP44	endoplasmic reticulum protein 44	+	yes		2.8
FASN	fatty acid synthase	+	yes		5.5
GALNT1	polypeptide N-acetylgalactosaminyltransferase 1	+	yes	TMP	only +HA
GLG1	golgi glycoprotein 1	+	yes	TMP	only +HA
GNA11	guanine nucleotide-binding protein subunit alpha-11	+	yes		only +HA
GNA13	G protein subunit alpha 13	++	yes		only +HA
GNAI1	guanine nucleotide-binding protein G(i) subunit alpha-1	++	yes		2.1
GNAS	guanine nucleotide-binding protein G(s) subunit alpha XLas-like isoform X1	++	yes		only +HA
H2AC8	H2A clustered histone 8	nd			11
HSD17B1	estradiol 17-beta-dehydrogenase 1	+	yes		4.1
IDH2	isocitrate dehydrogenase [NADP]. mitochondrial precursor	+	yes		2.5
IGSF8	immunoglobulin superfamily member 8	++	yes	TMP	only +HA
IMMT	inner membrane mitochondrial protein	+	yes	TMP	3.3
*ITGA6*	*integrin subunit alpha 6*	++	yes	TMP	510
*LDHC*	*lactate dehydrogenase C*	+	yes		*only +HA*
LMAN1	Lectin, mannose binding 1 (ERGIC-53)	+	yes	TMP	2.8
LSR	lipolysis stimulated lipoprotein receptor	+	yes	TMP	36
M6PR	mannose-6-phosphate receptor, cation dependent	++	yes	TMP	only +HA
MAN1B1	endoplasmic reticulum mannosyl-oligosaccharide 1.2-alpha-mannosidase	+	yes	TMP	only +HA
MBLAC2	metallo-beta-lactamase domain containing 2	+	yes		only +HA
MLEC	malectin	++	yes	TMP	5.9
MMP14	matrix metallopeptidase 14	++	yes	TMP	only +HA
MYOF	myoferlin	+	yes	TMP	39
NNT	nicotinamide nucleotide transhydrogenase	+	yes		7.5
OAT	ornithine aminotransferase	+	yes		9.7
PGK1	phosphoglycerate kinase 1	+	yes		2.6
PHB	prohibitin	+	yes		4.3
PI4K2A	phosphatidylinositol 4-kinase type 2-alpha	++	yes		only +HA
PLOD1	procollagen-lysine.2-oxoglutarate 5-dioxygenase 1	+	yes		only +HA
PLSCR2	phospholipid scramblase 1	predict		TMP	14
PLSCR3	phospholipid scramblase 3	+	yes	TMP	only +HA
PLXNB2	plexin-B2 precursor	+	yes	TMP	only +HA
PRAF2	PRA1 domain family member 2	+	yes		only +HA
PRDX6	peroxiredoxin 6	+	yes		only +HA
*PRR9*	*proline-rich protein 9*	*predict*			*only +HA*
PTBP1	polypyrimidine tract binding protein 1	++	yes		3.3
*PTGFRN*	*prostaglandin F2 receptor inhibitor*	+	yes	TMP	42
RAP2A	ras-related protein Rap-2a	++	yes		only +HA
ROR2	tyrosine-protein kinase transmembrane receptor ROR2	predict	yes	TMP	only +HA
RPN2	ribophorin II	+	yes		3.3
*S100A8*	*S100 calcium binding protein A8*	*predict*	yes		*only +HA*
*S100A9*	*protein S100-A9*	+	yes		*only +HA*
SCAMP2	secretory carrier membrane protein 2	+	yes		only +HA
SCAMP3	secretory carrier membrane protein 3	+	yes		only +HA
SCARB2	scavenger receptor class B member 2	+	yes		13
SCCPDH	saccharopine dehydrogenase-like oxidoreductase	+	yes		14
*SELENBP1*	*selenium-binding protein 1*	+	yes		*2.0*
SERPINH1	serpin family H member 1	+	yes		3.7
SIGMAR1	sigma non-opioid intracellular receptor 1	+	yes	TMP	only +HA
SLC44A1	choline transporter-like protein 1 isoform X1	++	yes		only +HA
SORT1	sortilin	+	yes	TMP	only +HA
STRA6	receptor for retinol uptake STRA6	predict	yes		only +HA
TMX1	thioredoxin related transmembrane protein 1	++	yes	TMP	6.4
TMX3	thioredoxin related transmembrane protein 3	+	yes	TMP	37
TMX4	thioredoxin related transmembrane protein 4	+	yes	TMP	14
TRFC	transferrin receptor protein 1 (p90, CD71)	nd	yes		only +HA
*TUBB4B*	*tubulin beta-4B chain*	++	yes		*3.56*
TUFM	Tu translation elongation factor, mitochondrial	+	yes		3.1
VAMP3	vesicle associated membrane protein 3	+	yes	TMP	only +HA
*VIM*	*vimentin*	+	yes		*3.73*

One plus (+) markes the proteins experimentally detected in human and/or bovine palmitoyl-proteomes according to SwissPalm database (https://swisspalm.org/, accessed on 17 March 2021) and/or published studies; two pluses (++) signify that palmitoylation of this protein was validated by different techniques; “predict”—signifies that palmitoylation on at least one Cys was predicted in this protein; nd—not detected in the datasets defined by selected parameters. Proteins, identified from bovine cumulus-oocytes complexes are presented in *italics*. Proteins, detected in one or more human and/or bovine exosome-proteomes according to Vesiclepedia database (http://www.microvesicles.org/, accessed on 21 January 2021) and/or published studies are notified by “yes”. TMP—transmembrane proteins, according to Membranome database (https://membranome.org/, accessed on 22 March 2021). Ratio of normalized weighted spectra mean values for each protein in +HA to −HA samples is shown (+HA enrichment fold).

**Table 2:** Analysis of cell component distribution of the proteins identified as palmitoylated in bovine follicular cells, using FunRich software [55].

Cellular Component	Number of Proteins (IDs)	Background (Number of Proteins)	Fold Enrichment	*p*-Value, BH Method
**Lysosome**	51	1620	6.0	1.14 × 10^−27^
ACAT1; AIFM1; APMAP; ATP1A1; CANX; CD36; CD81; CKAP4; CTNND1; DCD; DLAT; DNAJC5; ECE1; EPHX1; ERGIC3; ERP44; GLG1; GNA11; GNA13; GNAI1; GNAS; HSD17B1; IDH2; IGSF8; ITGA6; LMAN1; M6PR; MBLAC2; MLEC; MYOF; PGK1; PHB; PI4K2A; PLSCR3; PRDX6; PTBP1; PTGFRN; RAP2A; S100A8; S100A9; SCAMP2; SCARB2; SCCPDH; SIGMAR1; SLC44A1; SORT1; STRA6; TMX1; TMX4; TUBB4B; TUFM				
**Exosomes**	46	2043	4.3	5.46 × 10^−18^
ACAA2; ACAT1; ATP1A1; BLVRA; CANX; CD36; CD58; CD81; CKAP4; CTNND1; DCD; ECE1; ERP44; FASN; GLG1; GNA11; GNA13; GNAI1; GNAS; IGSF8; ITGA6; LMAN1; LSR; MBLAC2; MYOF; PGK1; PHB; PLOD1; PLSCR3; PLXNB2; PRDX6; PTBP1; PTGFRN; RAP2A; S100A8; S100A9; SCAMP2; SCAMP3; SCARB2; SELENBP1; SLC44A1; SORT1; TUBB4B; TUFM; VAMP3; VIM				
**Membrane**	15	350	8.1	9.22 × 10^−8^
APMAP; ATP1A1; CANX; CD81; CKAP4; GLG1; LMAN1; MLEC; MMP14; MYOF; RPN2; SCAMP2; SCAMP3; SCARB2; SLC44A1				
**Plasma membrane**	41	3479	2.2	4.09 × 10^−6^
ATP1A1; CANX; CD36; CD58; CD81; CKAP4; CRELD1; CTNND1; DNAJC5; ECE1; GLG1; GNA11; GNA13; GNAI1; GNAS; IGSF8; ITGA6; LDHC; LSR; M6PR; MMP14; MYOF; PHB; PLSCR2; PLSCR3; PLXNB2; PTGFRN; RAP2A; ROR2; S100A8; S100A9; SCAMP2; SCAMP3; SCARB2; SELENBP1; SERPINH1; SIGMAR1; SORT1; TMX1; VAMP3; VIM				
**Golgi apparatus**	20	897	4.2	4.08 × 10^−6^
CANX; CD36; CKAP4; DCD; ECE1; ERGIC3; FASN; GALNT1; GLG1; GNAI1; LMAN1; LSR; M6PR; PI4K2A; PTGFRN; SCAMP2; SCAMP3; SELENBP1; SORT1; VIM				
**Endoplasmic reticulum**	22	1104	3.8	4.35 × 10^−6^
CANX; CKAP4; CLGN; CRELD1; DNAJC5; EPHX1; ERP44; GALNT1; LMAN1; MAN1B1; MLEC; PI4K2A; PLOD1; PLXNB2; PTGFRN; RPN2; SERPINH1; SIGMAR1; SORT1; STRA6; TMX1; VIM				
**Mitochondrion**	22	1259	3.3	3.75 × 10^−5^
ACAA2; ACAT1; AIFM1; ALDH6A1; ATP1A1; CANX; DLAT; FASN; GLG1; IDH2; IMMT; LDHC; NNT; OAT; PGK1; PHB; PLSCR3; RPN2; SCCPDH; TMX1; TUFM; VIM				
**ER-Golgi intermediate compartment**	4	30	25.3	0.00175
ERGIC3; ERP44; LMAN1; SERPINH1				
**Cytoskeleton**	10	427	4.4	0.00647
CD36; CKAP4; DCD; IMMT; ITGA6; MLEC; PHB; TUBB4B; TUFM; VIM				
**Trans-Golgi network membrane**	2	3	126.2	0.00647
GNAS, SCAMP2				
**Recycling endosome membrane**	2	7	54.2	0.04065
RAP2A; SCAMP2				

## Data Availability

The mass spectrometry proteomics data have been deposited to the ProteomeXchange Consortium via the PRIDE partner repository [91], and are available on the site of PRIDE (https://www.ebi.ac.uk/pride/, accessed on 24 July 2020) with the dataset identifiers PXD020540 and PXD020547. Transcriptomic data are available via public repository Gene Expression Omnibus (GEO) with the accession number GSE149151.

## Supplementary Materials

The following are available online at https://www.mdpi.com/article/10.3390/ijms222111757/s1.

## Appendix B

**Table A1:** Differential analysis of expression of the enzymes involved in protein palmitoylation (GO:0018345) and depalmitoylation (GO:0002084, marked **in bold**) in bovine cumulus cells (CC) surrounding the oocyte before(immature) and after maturation in vivo, or in vitro (IVM). Mean values of relative expression of four independent samples from microarray data are shown for each gene. Different letters signify difference at *p* < 0.05 (ANOVA and post-hoc test).

*Gene*	Protein	Relative Expression		
		Immature	IVM	In Vivo Mature
***ABHD13***	**Lysophosphatidylserine lipase ABHD13**	78.71 (a)	271.11(b)	338.96 (b)
***GLUL***	**Glutamine synthetase**	1428.92 (ab)	1061.75 (a)	1568.31(b)
***GOLGA7***	**Golgin subfamily A member 7**	6662.79 (a)	7448.94 (a)	7398.62 (a)
***LYPLA1***	**Acyl-protein thioesterase 1**	8230.51 (a)	9801.03 (a)	10,408.22 (a)
***LYPLA2***	**Acyl-protein thioesterase 2**	43,732.30 (a)	35,366.24(ab)	30,134.65(b)
***LYPLAL1***	**Lysophospholipase-like protein 1**	230.95 (a)	65.20 (b)	229.32 (a)
***CLN1***	**Palmitoyl-protein thioesterase 1**	7213.40 (a)	1066.52 (b)	1802.02 (c)
***PPT2***	**Lysosomal thioesterase PPT2**	8166.68 (ab)	6956.55 (a)	9431.63 (b)
*SELK*	Selenoprotein K	12,928.36 (a)	17,856.50(b)	21,467.59(b)
*ZDHHC1*	Probable palmitoyltransferase ZDHHC1	31.00 (a)	18.98 (b)	13.08 (b)
*ZDHHC3*	Palmitoyltransferase ZDHHC3	4209.96 (a)	3597.84 (a)	5936.89 (b)
*ZDHHC4*	Palmitoyltransferase ZDHHC4	10,213.52 (a)	13,393.12(b)	15,747.39 (b)
*ZDHHC5*	Palmitoyltransferase ZDHHC5	1493.68 (a)	1844.64 (ab)	2200.70 (b)
*ZDHHC7*	Palmitoyltransferase ZDHHC7	2705.71 (a)	4347.91(b)	7287.39 (b)
*ZDHHC9*	Pralmitoyltransferase ZDHHC9	64.93 (a)	58.03 (a)	65.70 (a)
*ZDHHC12*	Probable palmitoyltransferase ZDHHC12	1646.18 (a)	2138.80 (b)	2400.89 (b)
*ZDHHC13*	Probable palmitoyltransferase ZDHHC13	1758.71 (a)	1542.11 (a)	2768.72 (b)
*ZDHHC15*	Palmitoyltransferase ZDHHC15	18.07 (a)	20.66 (a)	19.01 (a)
*ZDHHC16*	Palmitoyltransferase ZDHHC16	4788.19 (a)	4569.41 (a)	6682.16 (b)
*ZDHHC17*	Palmitoyltransferase ZDHHC17	1660.87 (a)	1197.13 (b)	1015.40 (b)
*ZDHHC18*	Palmitoyltransferase ZDHHC18	5589.22 (a)	7843.17 (b)	20,093.53 (c)
*ZDHHC19*	Palmitoyltransferase ZDHHC19	14.96 (a)	25.59 (b)	20.31 (b)
*ZDHHC20*	Palmitoyltransferase ZDHHC20	4469.49 (a)	5428.55 (a)	10,346.94 (b)
*ZDHHC21*	Palmitoyltransferase ZDHHC21	83.51 (a)	72.75 (a)	114.70 (a)
*ZDHHC22*	Palmitoyltransferase ZDHHC22	180.33 (a)	177.99 (a)	140.04 (a)
*ZDHHC24*	Probable palmitoyltransferase ZDHHC24	39,433.14 (a)	36,372.21 (a)	56,638.58 (b)
