# Supplementary material for: Protein Palmitoylation in Bovine Ovarian Follicle
Source: Int J Mol Sci. 2021 Oct 29;22(21):11757. doi: 10.3390/ijms222111757 (PMC8583988; doi:10.3390/ijms222111757)
Supplement: Supplementary file 1 [file ijms-22-11757-s001.zip › ijms-1396990-supplementary.pdf]

Supplementary data

Supplementary data Table S1. Gene expression of follicular palmitoyl-proteins in bovine granulosa cell, cumulus cells and oocyte, using microarray hybridization. Mean values of relative expression of four independent samples are shown for each gene. Different letters signify difference at p<0.05.

Gene	p-value	Granulosa cells	Cumulus cells	Oocyte
ACAA2	0.001	34.463 (a)	27.766 (a)	68.459 (b)
ACAT1	0.000	105.594 (b)	106.047 (b)	24.086 (a)
AIFM1	0.000	19.503 (a)	25.845 (a)	57.509 (b)
ALDH6A1	0.000	0.703 (b)	0.672 (b)	0.331 (a)
APMAP	0.000	12.230 (b)	23.661 (c)	1.428 (a)
ATP1A1	0.011	16.332 (a)	30.850 (b)	22.919 (ab)
BLVRA	0.000	75.982 (b)	130.174 (c)	1.068 (a)
CANX	0.003	233.852 (ab)	318.600 (b)	153.760 (a)
CD36	0.013	27.198 (b)	15.528 (ab)	3.883 (a)
CD58	0.000	1.949 (a)	2.501 (a)	53.007 (b)
CD81	0.773	13.256 (a)	18.177 (a)	22.398 (a)
CKAP4	0.030	674.865 (ab)	792.857 (b)	383.658 (a)
CLGN	0.008	24.413 (b)	9.976 (a)	16.893 (ab)
CRELD1	0.000	13.658 (b)	31.393 (c)	5.100 (a)
CTNND1	0.000	1.212 (a)	1.623 (a)	7.354 (b)
DNAJC5	0.000	3.799 (a)	3.958 (a)	7.958 (b)
ECE1	0.000	2.378 (b)	3.327 (c)	0.545 (a)
EPHX1	0.001	15.094 (a)	21.737 (b)	9.158 (a)
ERGIC3	0.000	248.992 (b)	339.813 (c)	34.344 (a)
ERP44	0.000	49.605 (a)	63.439 (a)	173.210 (b)
FASN	0.185	2.873 (a)	5.059 (a)	1.019 (a)
GALNT1	0.000	3.352 (a)	3.368 (a)	14.884 (b)
GLG1	0.000	34.213 (a)	76.984 (b)	24.007 (a)
GNA11	0.000	27.050 (a)	41.322 (a)	182.958 (b)
GNA13	0.000	0.811 (a)	0.954 (a)	3.615 (b)
GNAI1	0.000	1.357 (a)	1.169 (a)	14.376 (b)
GNAS	0.000	218.021 (b)	248.977 (b)	3.486 (a)
HSD17B1	0.000	873.825 (b)	2188 (c)	38.566 (a)
IDH2	0.000	77.683 (b)	64.868 (b)	6.077 (a)
IGSF8	0.000	4.317 (b)	10.195 (c)	0.559 (a)
IMMT	0.012	26.704 (a)	42.418 (b)	39.348 (b)
ITGA6	0.000	5.936 (b)	6.641 (b)	1.496 (a)
LDHC	0.000	0.726 (a)	0.719 (a)	95.821 (b)
LMAN1	0.008	9.436 (b)	11.057 (b)	4.985 (a)
LSR	0.000	16.312 (a)	23.688 (b)	38.136 (c)
M6PR	0.002	28.837 (a)	32.502 (a)	43.498 (b)
MAN1B1	0.004	3.153 (ab)	6.186 (b)	0.751 (a)
MBLAC2	0.000	2.111 (b)	1.956 (b)	0.262 (a)
MLEC	0.000	9.927 (a)	11.466 (a)	46.329 (b)

MMP14	0.004	1.067 (b)	0.882 (b)	0.452 (a)
MYOF	0.000	134.738 (b)	144.418 (b)	6.644 (a)
NNT	0.000	12.801 (b)	15.948 (b)	6.131 (a)
OAT	0.003	153.988 (a)	295.524 (b)	338.588 (b)
PGK1	0.000	344.460 (b)	853.946 (c)	35.570 (a)
PHB	0.037	20.212 (ab)	19.049 (a)	37.398 (b)
PI4K2A	0.000	0.509 (a)	0.578 (a)	0.930 (b)
PLOD1	0.000	4.076 (b)	21.899 (c)	0.290 (a)
PLSCR2	0.000	27.211 (b)	28.326 (b)	7.993 (a)
PLSCR3	0.018	28.339 (a)	32.621 (ab)	37.168 (b)
PLXNB2	0.000	64.740 (b)	86.194 (c)	5.360 (a)
PRAF2	0.000	56.951 (b)	83.633 (b)	1.923 (a)
PRDX6	0.001	67.287 (b)	61.864 (b)	36.694 (a)
PTBP1	0.000	131.658 (a)	175.859 (a)	330.625 (b)
PTGFRN	0.000	3.610 (b)	7.312 (c)	0.624 (a)
RAP2A	0.005	13.443 (ab)	17.690 (b)	9.237 (a)
ROR2	0.000	59.187 (b)	129.995 (c)	1.403 (a)
RPN2	0.001	110.448 (b)	144.970 (b)	37.114 (a)
S100A8	0.001	1.271 (b)	0.584 (a)	0.244 (a)
S100A9	0.000	0.666 (c)	0.501 (b)	0.246 (a)
SCAMP2	0.000	37.678 (a)	49.152 (a)	113.639 (b)
SCAMP3	0.000	126.553 (a)	154.982 (a)	385.571 (b)
SCARB2	0.000	576.420 (b)	578.115 (b)	29.324 (a)
SCCPDH	0.004	2.562 (a)	2.572 (a)	4.078 (b)
SELENBP1	0.000	2.442 (a)	1.445 (a)	179.307 (b)
SERPINH1	0.000	136.620 (b)	222.703 (c)	1.088 (a)
SIGMAR1	0.006	102.168 (ab)	152.670 (b)	65.402 (a)
SLC44A1	0.000	0.530 (b)	0.540 (b)	0.306 (a)
SORT1	0.000	1.795 (b)	3.318 (c)	0.834 (a)
STRA6	0.000	1164 (b)	2488 (c)	23.725 (a)
TMX1	0.003	17.581 (b)	16.391 (b)	8.296 (a)
TMX3	0.000	1.835 (a)	1.638 (a)	6.749 (b)
TMX4	0.000	3.594 (b)	3.952 (b)	1.233 (a)
TUBB4B	0.003	613.015 (a)	1101 (ab)	1528 (b)
TUFM	0.000	150.854 (a)	184.109 (a)	383.048 (b)
VAMP3	0.000	92.392 (a)	104.297 (a)	284.722 (b)
VIM	0.000	913.989 (b)	1235 (c)	7.346 (a)

Supplementary data Table S2. Expression of the enzymes involved in protein palmitoylation (GO:0018345) and depalmitoylation (GO:0002084, marked **in bold**) in bovine oocytes (Oo), cumulus cells (CC) and granulosa cells (GC) using microarray hybridisation. Mean values of relative expression of four independent samples are shown for each gene. Different letters signify difference at p<0.05.

Gene	Gene/product name	*p-value*	Relative expression		
			Oo	CC	GR
ABHD12	**Lysophosphatidylserine lipase ABHD12**	0.51	112.1 (a)	186.5 (a)	184.2 (a)
ABHD13	**Protein ABHD13**	<0.01	1.2 (a)	4.7 (c)	4.0 (b)
ABHD17A	**Alpha/beta hydrolase domain-containing protein 17A**	0.171	84.9 (a)	27.9 (a)	23.526 (a)
ABHD17C	**Alpha/beta hydrolase domain-containing protein 17B**	<0.01	72.3 (b)	7.6 (a)	11.3 (a)
CLIP3	CAP-Gly domain-containing linker protein 3	0.079	0.9 (a)	1.2 (a)	1.072 (a)
CLN1	**Palmitoyl-protein thioesterase 1**	<0.01	11.8 (a)	159.1 (b)	151.3 (b)
GLUL	Glutamine synthetase	<0.01	333.5 (b)	28.9 (a)	39.1 (a)
GOLGA7	Golgin subfamily A member 7	<0.01	403.3 (b)	35.6 (a)	30.6 (a)
GOLGA7B	Golgin subfamily A member 7B	<0.01	88.0 (b)	0.6 (a)	0.6 (a)
HHATL	Protein-cysteine N-palmitoyltransferase HHAT-like	<0.01	3.2 (b)	0.5 (a)	0.6 (a)
LYPLA1	**Acyl-protein thioesterase 1**	<0.01	2.2 (a)	31.6 (b)	29.2 (b)
LYPLA2	**Acyl-protein thioesterase 2**	<0.01	9.8 (a)	99.6 (b)	101.7 (b)
LYPLAL1	**Lysophospholipase-like protein 1**	<0.01	14.1 (b)	1.8 (a)	1.8 (a)
MAP6D1	MAP6 domain-containing protein 1	<0.01	1.3 (a)	5.9 (b)	5.2 (b)
PPT2	**Lysosomal thioesterase PPT2**	0.11	99.2 (a)	90.9 (a)	67.7 (a)
SELK	Selenoprotein K	<0.01	285.4 (b)	76.7 (a)	63.1 (a)
ZDHHC1	Probable palmitoyltransferase ZDHHC1	<0.01	9.4 (b)	8.8 (b)	4.2 (a)
ZDHHC2	Palmitoyltransferase ZDHHC2	<0.05	6.3 (ab)	5.0 (a)	7.9 (b)
ZDHHC3	Palmitoyltransferase ZDHHC3	<0.01	64.0 (b)	19.8 (a)	16.9 (a)
ZDHHC4	Probable palmitoyltransferase ZDHHC4	<0.01	44.7 (a)	110.1 (b)	93.7 (b)
ZDHHC5	Palmitoyltransferase ZDHHC5	<0.01	37.8 (b)	19.3 (a)	17.9 (a)
ZDHHC6	Palmitoyltransferase ZDHHC6	<0.01	71.6 (b)	28.21 (a)	22.8 (a)
ZDHHC7	Palmitoyltransferase ZDHHC7	<0.01	3.1 (b)	0.9 (a)	1.0 (a)
ZDHHC8	Probable palmitoyltransferase ZDHHC8	0.41	0.7 (a)	0.5 (a)	0.5 (a)
ZDHHC9	Palmitoyltransferase ZDHHC9	<0.01	5.6 (a)	42.2 (c)	28.3 (b)
ZDHHC12	Probable palmitoyltransferase ZDHHC12	0.069	5.8 (a)	1.8 (a)	1.5 (a)
ZDHHC13	Probable palmitoyltransferase ZDHHC13	<0.01	38.0 (a)	3.1(b)	4.5 (b)
ZDHHC14	Probable palmitoyltransferase ZDHHC14	<0.01	0.7 (a)	91.2 (c)	68.3 (b)
ZDHHC15	Palmitoyltransferase ZDHHC15	0.391	0.26 (a)	2.546 (a)	0.7 (a)
ZDHHC16	Palmitoyltransferase ZDHHC16	<0.01	82.8 (b)	54.6 (a)	42.7 (a)
ZDHHC17	Palmitoyltransferase ZDHHC17	<0.01	0.43 (a)	8.1 (b)	8.5 (b)
ZDHHC18	Palmitoyltransferase ZDHHC18	<0.01	1.5 (b)	0.7 (a)	0.6 (a)
ZDHHC19	Palmitoyltransferase ZDHHC19	<0.01	5.6 (b)	2.3 (a)	2.2 (a)
ZDHHC20	Palmitoyltransferase ZDHHC20	<0.05	3.7 (ab)	3.1 (a)	4.7 (b)
ZDHHC21	Palmitoyltransferase ZDHHC21	<0.01	5.5 (a)	21.6 (c)	11.7 (b)
ZDHHC22	Palmitoyltransferase ZDHHC22	<0.01	0.2 (a)	0.4 (b)	0.5 (b)
ZDHHC23	Palmitoyltransferase ZDHHC23	<0.01	0.9 (b)	0.6 (a)	0.8 (a)
ZDHHC24	Probable palmitoyltransferase ZDHHC24	0.501	0.5 (a)	0.6 (a)	0.6 (a)

Supplementary data, Table S3. List of primers used for real time PCR analysis of gene expression in bovine follicular cells

Gene	Accession	Primer	Séquence 5'-3'	Amplicon (bp)	Efficiency (%)
CLN1	NM_174154	fw	TTACGGCTGTCCTCCTCTGA	293	99.4
		rev	AAGGGACAGGTTTATCAAGGGT		
PPT2	NM_001035318	fw	GCAACTACTGGCATGACCCT	263	95.3
		rev	CCCAAAAGAGTCCCGCAGAT		
GAPDH	NM_001034034	fw	TTCAACGGCACAGTCAAGG	119	100.3
		rev	ACATACTCAGCACCAGCATCAC		
RPL19	NM_001040516	fw	AATCGCCAATGCCAACTC	156	99.9
		rev	CCCTTTCGCTTACCTATACC		
SDHA	NM_174178	fw	GCAGAACCTGATGCTTTGTG	185	89.2
		rev	CGTAGGAGAGCGTGTGCTT		

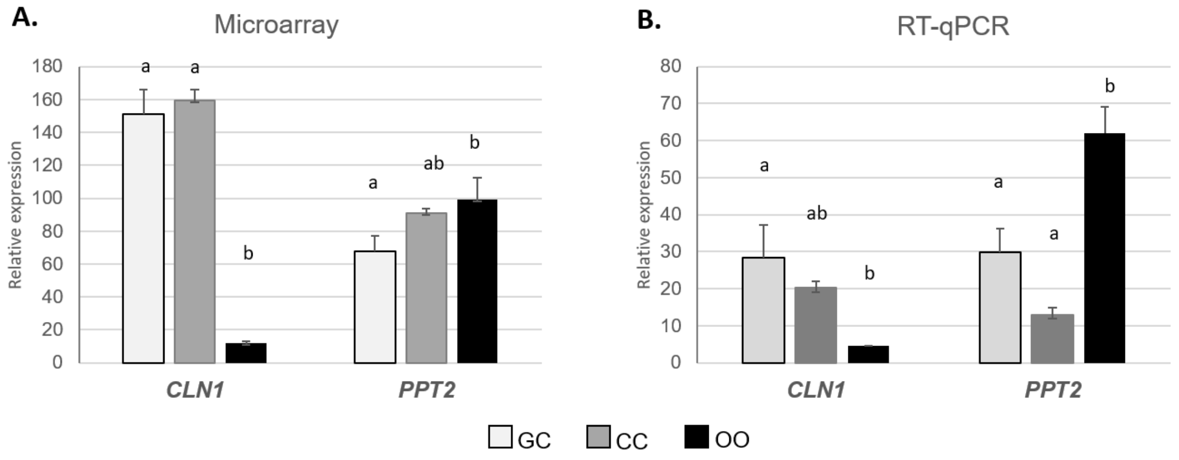

Supplementary Figure S1. Real time PCR validation of microarray gene expression analysis in bovine granulosa cells (GR), cumulus cells (CC) and oocytes. *CLN1* - palmitoyl-thioesterase 1 (PPT1), *PPT2* - palmitoyl-thioesterases 2. **A.** Relative expression of *CLN1* and *PPT2* genes as analysed by microarray (GSE149151). Histogram bars are the mean values of four independent samples +/- SEM. **B.** Relative expression of *CLN1* and *PPT2* analysed by real-time PCR. Histogram bars are the mean normalized values of six independent samples +/- SEM.
